# Do Salient Social Norms Moderate Mortality Salience Effects? A (Challenging) Meta-Analysis of Terror Management Studies

**DOI:** 10.1177/10888683221107267

**Published:** 2022-08-11

**Authors:** Simon Schindler, Joe Hilgard, Immo Fritsche, Brian Burke, Stefan Pfattheicher

**Affiliations:** 1 University of Kassel, Germany; 2 Illinois State University, Normal, USA; 3 University of Leipzig, Germany; 4 Fort Lewis College, Durango, CO, USA; 5 Aarhus University, Denmark

**Keywords:** terror management theory, mortality salience, social norms, meta-analysis, publication bias

## Abstract

Terror management theory postulates that mortality salience (MS) increases the motivation to defend one’s cultural worldviews. How that motivation is expressed may depend on the social norm that is momentarily salient. Meta-analyses were conducted on studies that manipulated MS and social norm salience. Results based on 64 effect sizes for the hypothesized interaction between MS and norm salience revealed a small-to-medium effect of *g* = 0.34, 95% confidence interval [0.26, 0.41]. Bias-adjustment techniques suggested the presence of publication bias and/or the exploitation of researcher degrees of freedom and arrived at smaller effect size estimates for the hypothesized interaction, in several cases reducing the effect to nonsignificance (range *g*_corrected_ = −0.36 to 0.15). To increase confidence in the idea that MS and norm salience interact to influence behavior, preregistered, high-powered experiments using validated norm salience manipulations are necessary. Concomitantly, more specific theorizing is needed to identify reliable boundary conditions of the effect.

Terror management theory (TMT; Greenberg et al., 1986) postulates that people deal with their mortality by defending and living up to their cultural worldviews. In line with the focus theory of normative conduct (Cialdini et al., 1991), a substantial body of experimental studies has provided support for the idea that reactions to mortality salience (MS) depend on the salience of social norms, providing a possible explanation for seemingly contradictory findings in TMT research (e.g., Jonas et al., 2008). To review evidence for this idea, we conducted a meta-analysis of studies that experimentally manipulated MS and social norm salience. Given that social norms define the cultural part of one’s worldview, this idea reflects the original cultural worldview defense hypothesis of TMT and concurrently addresses the predictability of MS reactions. Because this idea also poses substantial implications for understanding and predicting (destabilizing) societal dynamics in the face of existential threats such as terrorist attacks, assessing the empirical evidence would prove valuable, especially considering the replication crisis in the field of social psychology (Open Science Collaboration, 2015).

## Basic Propositions of TMT

TMT (Greenberg et al., 1986) is based on the work of cultural anthropologist Ernest Becker. He proposed that culture is important for the assurance of worth and safety when confronted with the awareness of one’s own death (Becker, 1972). If this cultural endorsement is lacking, a paralyzing anxiety arises, resulting from what Becker termed the “terror of death.” To cope with this terror, TMT posits that it is necessary to maintain self-esteem—that is, the “sense that we are valuable parts of a meaningful, important, and enduring existence” (Solomon et al., 1991, p. 106). The proposed cultural anxiety buffer thus consists of two interrelated components: first, faith in a culturally validated worldview that gives meaning and purpose to human life, along with faith in the provided norms and standards that specify which behavior is valued in this certain worldview, and second, the belief that one is meeting or exceeding these norms and standards. Thus, certainty about the validity of one’s cultural worldview and one’s value within a culture is crucial for the effectiveness of the death anxiety-buffering system. Because there are different worldviews and subjective evaluation criteria for meeting the social norms and standards within a culture, people are motivated to continually have others consensually validate their worldview and self-esteem.

## The Mortality Salience Hypothesis

Although different hypotheses derived from TMT have been tested, most research addressed the MS hypothesis stating that being confronted with their own mortality increases people’s need for the protection provided by their cultural worldview and self-esteem (Burke et al., 2010; Pyszczynski et al., 2015). Consequently, MS is predicted to lead to more positive responses to anyone or anything that bolsters one’s cultural worldview/self-esteem and more negative responses to anyone or anything that threatens it. Support for this hypothesis emerges from empirical studies conducted in more than 20 countries on at least five continents around the globe, showing that MS increases motivation to enhance and defend diverse aspects of these components of the cultural anxiety buffer (Routledge & Vess, 2019). In the first empirical investigation of the MS hypothesis, for example, MS increased punishment toward a person who violated important worldview aspects (a prostitute); on the other hand, MS increased support for a person who acted in line with these aspects (Rosenblatt et al., 1989).

From the perspective of TMT, social norms constitute a fundamental part of our cultural worldviews (Becker, 1962, 1972; Berger & Luckmann, 1967; Goffman, 1959) and provide an orderly symbolic reality that allows people to view themselves as meaningful if they live up to those norms (e.g., Greenberg et al., 1997). Thus, MS should increase adherence to social norms because doing so would provide a source of both self-esteem and cultural worldview validation.

## The Significance of Social Norms

Social norms systematically and powerfully influence human behavior (e.g., Cialdini et al., 1991). For example, social norms direct us to congratulate people on their birthdays or give presents for Christmas (in some cultures), and they proscribe that we shout at our supervisors or talk badly about recently deceased people.

Due to the popularity of the study of social norms across different research fields, there exists significant variation in what constitutes a social norm (Hogg, 2010; Horne & Mollborn, 2020; Legros & Cislaghi, 2020). Differences exist about what it means for a norm to be *social* (e.g., that the interaction partners are human; that norms carry expectations from other people; that they hold social meaning; that they pose order and structure on society or mark group prototypes and boundaries). There are also different levels of analysis: Some researchers (e.g., in the field of sociology) conceptualize social norms as collective constructs—behavioral regularities of a social phenomenon on the group level. Other researchers (e.g., in psychological science) conceptualize social norms with a focus on the person instead—that is, individual perceptions about what others do and what others expect (Legros & Cislaghi, 2020).

In the present work, we apply a person-centered definition of social norms as we examine the anxiety-buffering role of social norms on the individual level. We follow Cialdini and Trost (1998, p. 152) who define social norms as “rules and standards that are understood by members of a group, and that guide and/or constrain social behavior.” Accordingly, social norms can tell the individual what others commonly do (i.e., *descriptive norms*) as well as what others commonly approve or disapprove of (i.e., *injunctive norms*). That is, descriptive norms refer to information about what most members of a group are doing in a given situation (Goldstein & Cialdini, 2007; Hogg & Reid, 2006). In contrast, injunctive norms can be regarded as shared rules of a certain group about how one should behave. Norms differ according to how internalized they are (e.g., Ajzen & Fishbein, 1970; Schwartz, 1977). Some norms become so internalized that they constitute *personal norms*. These reflect values that serve as guiding principles in people’s lives and self-expectations regarding behavior (Schwartz, 1977, 1992; Schwartz & Bilsky, 1990).

Given that existing norms can prove contradictory (e.g., the norm of minding one’s own business vs. the norm of getting involved; Cialdini, 2012), a question arises around which of the applicable norms will guide behavior in a situation. To address this question, Cialdini and colleagues developed the *focus theory of normative conduct*. The theory claims that norms only direct behavior when they are chronically accessible or salient in a particular situation (Cialdini, 2012; Cialdini & Trost, 1998). This idea was tested and supported in various contexts, showing that norm activation increased the likelihood of norm-compliant behavior. For example, in a study by Cialdini et al. (1990), participants stood in a clean parking area (i.e., anti-littering norm is present) or in a littered parking area (i.e., pro-littering norm is present). In half of the instances, a confederate additionally dropped a flier on the floor, whereas in the other half, the confederate just walked by. Seeing the confederate littering should draw the participants’ attention to the present norm (anti-littering vs. pro-littering norm). In line with the theory, participants littered more in the already littered parking area than in the clean parking area, and this effect was amplified when norm salience was high (i.e., when the confederate dropped a flier).

The focus theory of normative conduct draws on the priming principle, meaning that a triggering stimulus in the environment can influence subsequent reactions by automatically making more accessible mental representations connected to this stimulus (for reviews, see Bargh, 1997; Janiszewski & Wyer, 2014). That is, like every cognitive construct (e.g., E. T. Higgins & Bargh, 1987), norms must be salient in attention or high in accessibility to influence behavior.

## Specifying Predictions of MS Effects: The Role of Norm Salience

Cultural worldviews are not simple uniform constructs but rather prescribe a complex set of social norms and values that can be contradictory (Schwartz, 1992). Correspondingly, earlier studies showed that MS increased helping (Jonas et al., 2002), tolerance (Greenberg et al., 1992), and forgiveness and compassion (Schimel et al., 2006; Vail et al., 2009); conversely, MS also promoted aggression (McGregor et al., 1998; Pyszczynski et al., 2006), punishment (Rosenblatt et al., 1989), materialism, and accumulation of personal wealth (Kasser & Sheldon, 2000). In light of the vast number of different reactions to MS, TMT has been criticized for being unfalsifiable because any finding could be interpreted as evidence in favor of the theory, bringing up the question “to what elements of their cultural worldviews will people be reacting?” (L. L. Martin & van den Bos, 2014, p. 40). In other words, if MS increases the motivation to defend and live up to one’s worldview, which part of this worldview actually guides people’s reactions to MS?

One solid answer to this problem emerges from the following research. To explain diverse and sometimes opposite effects of MS and to better predict people’s reaction to MS, Jonas and colleagues (2008) combined TMT with the focus theory of normative conduct (Cialdini et al., 1991), proposing that “the norm that influences action following MS should be the one that is most prominent in consciousness at the moment” (p. 1241). In an early study, Greenberg et al. (1992) provided initial support for this idea by showing that activating the concept of tolerance reduced the tendency to devaluate different others after MS. To further test this idea, Jonas et al. (2008) conducted several studies in which they primed participants with different norms and manipulated MS. In one experiment, for example, MS increased participants’ pacifistic attitudes but only when the concept of pacifism was first activated through a word-search puzzle manipulation. A further experiment showed that MS increased participants’ suggested bonds for a woman arrested for illegal prostitution only when the concepts of security and conservatism (vs. benevolence and universalism) had been previously activated. Thus, from a TMT perspective, the work of Jonas et al. (2008) makes an important theoretical point: In a social world of shifting and sometimes opposing standards, concerns about mortality increase reactions corresponding to that concept which is most accessible in a given situation. This may result in increased self-interest, harsh punishment, or aggression; conversely, it may result in increased leniency, peace-making, or prosociality.

In the past decade, many additional experiments have been published, providing evidence for the idea regarding various additional social norms, such as helping and responsibility (Gailliot et al., 2008), honesty (Schindler & Reinhard, 2015a; Schindler, Reinhard, et al., 2019), modesty (Du & Jonas, 2015), justice and fairness (Hirschberger et al., 2016; Jonas et al., 2013), tolerance (Greenberg et al., 1992; Vail et al., 2019), individualism and collectivism (Courtney et al., 2021; Giannakakis & Fritsche, 2011; Jonas & Fritsche, 2012), religious norms (Rothschild et al., 2009; Schumann et al., 2014), pro-environmental norms (Fritsche et al., 2010; Harrison & Mallett, 2013), and the norm of reciprocity (Schindler et al., 2012, 2013). However, to date, no meta-analysis has quantified the established interaction effect between MS and social norm salience.

By focusing on social norms, the present work aims to assess the evidence for the *cultural* aspects of people’s worldview. When reviewing the literature, we realized that a specific social norm is sometimes not explicitly referred to as such; instead, the primed worldview concepts often fall into the additional category of personal norms (i.e., internalized social norms), in the sense of social values that serve as guiding principles in people’s lives and self-expectations for how to behave (Schwartz, 1992). “Tolerance,” for example, can be conceptualized and operationalized as a personal but also a social norm. In this regard, we also took studies into account that manipulated the salience of personal norms but which could also be considered a social norm (e.g., tolerance, magnanimity, and compassion). Hence, we applied a broad conceptualization of social norms—having the additional benefit of including a larger number of studies in our meta-analysis. With this broad conceptualization of social norms, it can be difficult to code studies as meeting or not meeting these definitions, and some judgment calls were necessary. We strove to be transparent as possible regarding our inclusion criteria and inclusion decisions and to report results when using both a narrow and a more liberal study selection.

## Meta-Analytical Evidence and Replicability of MS Effects

Meta-analysis provides a powerful and comprehensive tool for assessing the size of effects across research studies. In the most comprehensive MS meta-analysis to date (*k* = 277), Burke et al. (2010) reported a moderate to strong effect of MS (*d* = 0.75) across diverse aspects of the anxiety buffer. However, one pervasive problem in the social sciences, including psychology, that threatens the accuracy of meta-analytic estimates is publication bias (Bakker et al., 2012; Franco et al., 2014, 2016)—that is, the tendency of authors to submit, and journals to publish, studies with statistically significant as opposed to nonsignificant results. This can lead to overestimation of effects, as studies yielding large, significant effects are published and recovered for meta-analysis, whereas studies yielding null or countertheoretical results go undiscovered. Another related threat is researcher degrees of freedom—the possibility for researchers to analyze data in multiple ways and report a subset of analyses that yield statistically significant results. These causes of overestimation can dramatically increase Type I error rates in the meta-analysis (Sterling, 1959).

Several statistical techniques have been developed to identify and adjust for these biases (e.g., Carter et al., 2019). A corresponding reanalysis of the data by Burke et al. (2018) found signs of publication bias; a conservative adjustment estimated *d* = 0.32 and a more liberal adjustment estimated *d* = 0.61 as the true effect size of MS manipulations (see also Rodríguez-Ferreiro et al., 2019).

The number of studies on the MS hypothesis has since increased to more than 1,000 (L. Chen et al., 2022). Given that most of these studies applied the classic MS paradigm (see Schindler et al., 2021), many MS effects on worldview defense can thus be seen as conceptually replicated. On the other hand, preregistered and high-powered studies on MS effects are rare (for exceptions, see Courtney et al., 2021; Dunn et al., 2020; Schindler, Pfattheicher, et al., 2019; Vail et al., 2019). Interestingly, two registered reports were recently published, and they aimed to replicate earlier MS effects. Rodríguez-Ferreiro et al. (2019; 64 participants per cell) failed to replicate a study by Goldenberg et al. (2001; 10 participants per cell) testing the idea that participants under MS should react with more positive evaluations of an essay describing humans as distinct from animals. Schindler et al. (2021) tested the validity of the worldview defense hypothesis with conceptual replications. In two lab studies and one highly powered online study to detect even small effects (*N* = 1,356), no evidence was found for the expected MS effects. An internal meta-analysis revealed a small, nonsignificant effect of MS. Another work refers to a large-scale replication project called Many Labs 4 (Klein et al., 2022). Across 17 labs (*N* = 1,550), the authors report no significant MS effect on a classic measure of worldview defense, although the validity of this finding is disputed (Chatard et al., 2020; for a Bayesian reanalysis, see Haaf et al., 2020). In any case, the above-mentioned failures to replicate MS effects on worldview defense measures point to the informative value of detecting potential selection biases in the literature.

## The Present Meta-Analysis

**Figure 1.**
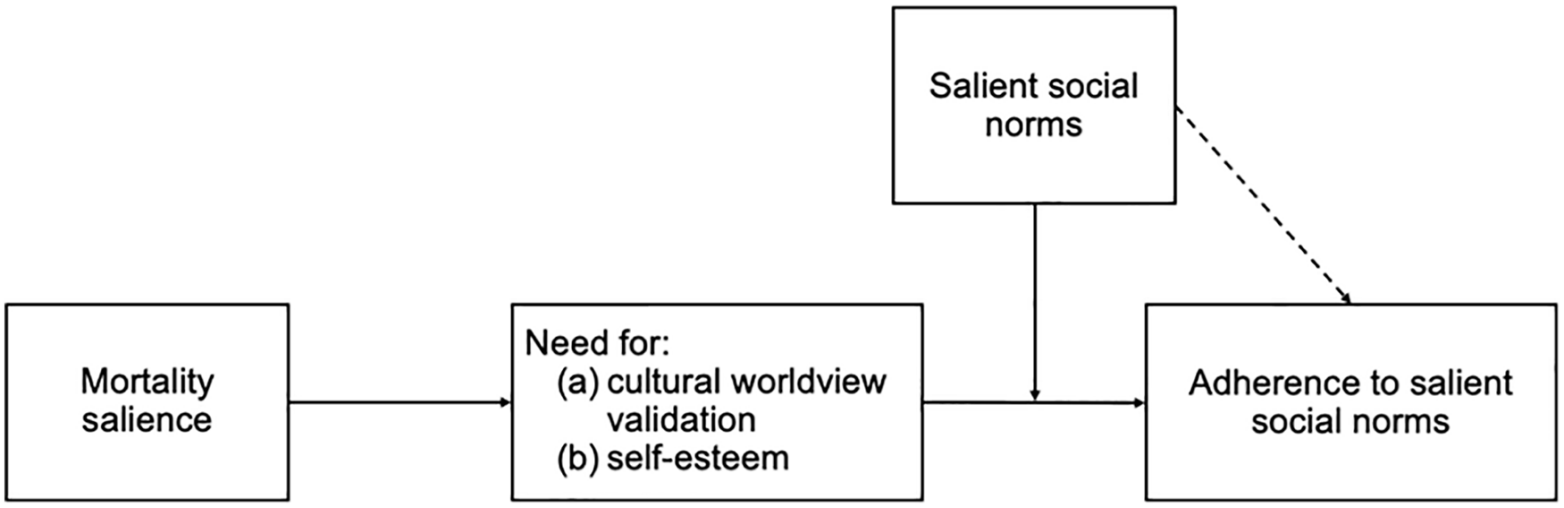
Theoretical model of the tested interaction hypothesis between mortality salience and social norm salience. *Note.* It is assumed that mortality salience increases the need for cultural worldview validation and self-esteem. To fulfill these needs, it is hypothesized that subsequent reactions depend on the situational salience of social norms. It is further expected that norm priming has an effect on adherence to salient social norms independent from MS (see dashed arrow).

The original MS hypothesis states that people under MS defend and live up to their *cultural* worldview. The present work aims to assess the evidence for the *cultural* aspects of people’s worldview, namely, to what extent people will defend or bolster their valued social norms. As depicted in Figure 1, MS increases the need for cultural worldview validation and self-esteem. To fulfill these needs, it is hypothesized that subsequent reactions depend on the situational salience of specific social norms. Correspondingly, the focus of our meta-analysis is on testing the interaction effect between MS and social norm salience. We therefore exclusively focused on studies that manipulated both MS and norm salience. With the present data set, we test norm priming effects with and without MS (see dashed arrow in Figure 1) and contribute to the assessment of social norm priming effects.

Assessing the empirical validity through meta-analysis and inspection for publication bias seems advisable, given the lack of preregistered replication attempts on this idea and given that recent replication attempts of other MS findings have not been uniformly successful (Rodríguez-Ferreiro et al., 2019; Schindler et al., 2021). We further believe that investigating this hypothesis is especially important because it addresses the previously discussed issue of the potential nonfalsifiability of TMT. The MS × Norm Salience hypothesis allows a clear a priori prediction—that is, MS increases reactions in accordance with the salient norm. Accordingly, since reversed findings are evidence against TMT (e.g., if MS increases reactions as opposed to the salient norm), there is an obvious criterion for falsification.

All datasheets, R codes, documentation of inclusion decisions, and further material can be found on the Open Science Framework (OSF) (https://osf.io/mr4nb/?view_only=8fac693905be4138a905f9b2b30cab6c)

## Method

### Inclusion Criteria

To be included in the present analyses, studies had to fulfill the following criteria:

1. Studies had to apply a generic experimental manipulation of MS (vs. mortality-not-salient).
2. Studies had to apply an experimental manipulation of the salience of a *specific* social norm (the second experimental condition may refer to no norm salience or opposed norm salience, such as salience of pro- vs. anti-environmental norms or individualism vs. collectivism). However, the various conceptualizations and operationalizations in the TMT literature do not allow a clear distinction between when a primed concept is a social norm and when it is a personal norm. To include as many studies as possible, we relied on a broad operationalization of norms. That is, we included articles investigating descriptive norms (providing information about what is commonly done) and injunctive norms (providing information about what should be done). We further included articles investigating personal norms (social values that serve as guiding principles in people’s lives and self-expectations of how to behave). To address potential differences among these three norm categories, we exploratively investigated this factor as a moderator. Again, it should be noted that applying a broad conceptualization of social norms can lead to difficulties in coding studies and in determining eligibility; thus, judgment calls were likely.
3. In TMT research, researchers have investigated dispositional variables that potentially reflect chronic accessibility of worldview relevant aspects. However, we focused on situational (i.e., manipulated) social norm salience. Therefore, studies had to be designed to test an interplay between the MS and the norm salience manipulation. Nevertheless, we also included studies testing a moderation of the MS × Norm Salience interaction (i.e., by including a third manipulated factor or a measure of individual differences).
4. Studies had to measure reactions (attitudes, intentions, and actual behavior) that relate to the manipulated norm and reflect direct norm compliance as a way to cope with MS. For this reason, we did not include studies using manipulations aiming to induce high self-esteem, self-affirmation, secure attachment, self-transcendence, or other states that had been hypothesized to buffer against MS. Self-affirmation, for example, is assumed to have the salutary effect of making people secure toward threatening events (Steele, 1988), that is, self-affirmation manipulations already refer to manipulating *adherence* to personal norms and values (e.g., Schmeichel & Martens, 2005). Given that we were interested in studies that prime specific norms and assess norm compliance to cope with MS, we decided to exclude studies that experimentally induced a buffering, secure state via affirming norms and values.
5. Sufficient data for the calculation of an effect size had to be reported in the respective article or provided by the authors.

### Literature Search

**Figure 2.**
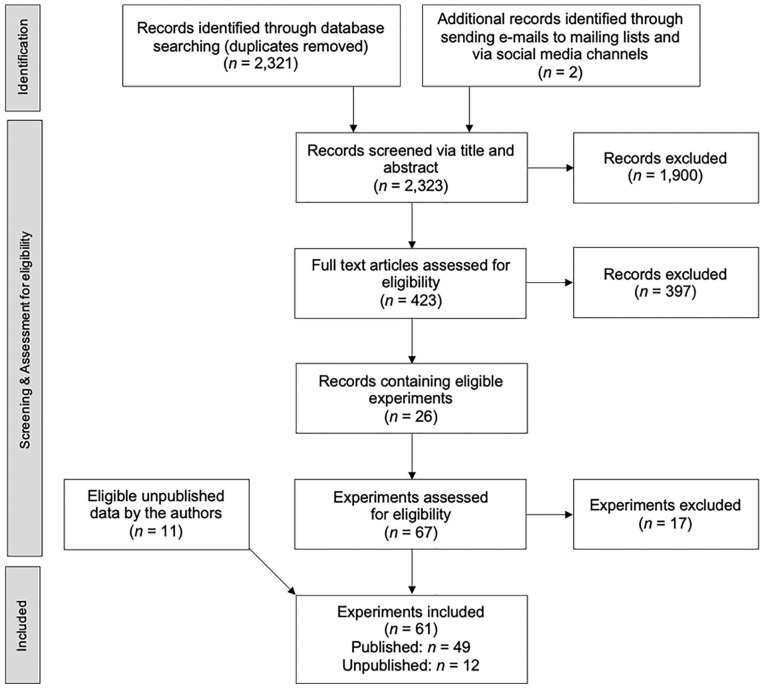
PRISMA flow diagram showing selection of studies for the present meta-analysis.

Figure 2 depicts a flow diagram showing the process of study selection. Several methods were used to detect literature that satisfied the inclusion criteria. First, we conducted a database search with PsycINFO, PsycARTICLES, and Web of Science using the following search string: (“mortality salien*” OR “mortality reminder*” OR “terror management” OR “existential threat”) AND (norm OR norms OR normativ OR conformity OR compliance OR adherence OR value* OR belief* OR violen* OR prosocial* OR materialis* OR prim* OR moderat* OR prescri*). After excluding duplicates, these searches yielded 2,321 records.

We called for unpublished data via the mailing lists of the European Association of Social Psychology, the German Psychological Association, and the TMT mailing list (including more than 30 TMT researchers). We posted requests in different online discussion forums of the Society of Personality and Social Psychology (SPSP) and in the Facebook group Psychological Methods Discussion Group (more than 40,000 members). The search for unpublished data was terminated on June 2, 2021. These requests generated two additional studies, none of which fit our inclusion criteria. Searching for relevant preprints on PsyArXiv using the keyword “mortality salience” yielded no eligible experiments.

Next, we screened these 2,323 records via titles and abstracts, leaving us with 423 potential records. A list of these 423 records can be found on the OSF. We assessed the eligibility of these records by reviewing the full texts and identified 26 records that reported at least one relevant experiment.^1^ In sum, these 26 records included 50 eligible experiments. Data from 11 unpublished studies that satisfied the inclusion criteria were provided by the first and the third author. In total, we identified 61 eligible experiments (49 published, 12 unpublished).

### Effect Size Extraction

Effect sizes were extracted as standardized mean differences (Hedges’s *g*; Hedges, 1981) and their standard errors^2^ using Excel spreadsheets and R scripts. An overview of calculation specifics can be found on the OSF. Five different sets of mean effect sizes were extracted and meta-analyzed: (a) the MS × Norm Salience interaction; (b) the contrast between MS and mortality-not-salient conditions with a salient norm; (c) the contrast between mortality-salient and mortality-not-salient conditions with no salient norm; (d) the contrast between norm-salient and norm-not-salient conditions when mortality was made salient; and (e) the contrast between norm-salient and norm-not-salient conditions when mortality was not made salient. In some studies, the salience of two opposing norms was manipulated; in these cases, contrasts between MS and mortality-not-salient conditions were extracted for both norms. In addition to the manipulation of two opposing norms, some studies also included neutral norm salience conditions. In these cases, effect sizes for two MS × Norm salience interactions were calculated while modeling their dependency. Some studies did not feature a condition without a salient norm and were therefore only included for the interaction terms and contrasts between MS and mortality-not-salient conditions given a salient norm.

In some cases, MS was hypothesized to attenuate or diminish a certain behavior. In these studies—without norm priming—MS was expected, for example, to enhance derogation of an anti-American author by American participants (Greenberg et al., 1994). By priming tolerance, these reactions should be attenuated so that there is no effect of MS compared with the control condition. Due to this predicted null effect of MS (vs. control condition), it was not appropriate to extract simple effects of MS in these cases; however, within the MS condition, there should be an effect of priming tolerance, such that derogation of worldview-conflicting stimuli is attenuated in the tolerance prime condition (compared with a priming control condition). This effect can be explained by adherence to tolerance. Thus, these studies and their interactions are relevant for our meta-analysis as the interaction pattern reflects norm compliance.

Some missing data were provided by the authors of the respective papers. When cell sizes were unavailable, we assumed equal cell sizes across conditions. If the necessary summary statistics to calculate effect size were unavailable, effect size was estimated from *t*- and χ^2^-values (e.g., Borenstein, 2009; Lipsey & Wilson, 2001) using the compute.es package for R (Del Re, 2013). In several studies not reporting means and *SDs*, we extracted cell means from the figures presented in the respective article using the software WebPlotDigitizer (Rohatgi, 2020). In this case, corresponding standard errors were calculated using the reported *F*-tests.

In some studies, several dependent variables were assessed; here, we chose the one closest to actual behavior (defined as a decision with real consequences). If none of the dependent variables referred to intention or behavior but only attitudes, we chose the one that was assessed first.

In some studies, a three-way interaction was investigated, that is, a third variable was predicted to moderate the MS × Norm Salience interaction. In these cases, we calculated the effect size of the interaction for the condition or trait level for which the interaction was predicted. This was a pragmatic decision because there was often insufficient information provided for calculating the effect size for the two-way interaction across the third variable. In three cases, however, we used the effect size for the two-way interaction across the third variable because no other information was provided.

The final number of included effect sizes for the interaction effect was *m* = 64 (61 studies; *N* = 8,195). The number of included effect sizes was *m* = 71 (56 studies) for the contrast between MS and mortality-not-salient in the norm-salient condition, *m* = 36 (36 studies) for the contrast between MS and mortality-not-salient in the norm-not-salient condition, *m* = 39 (36 studies) for the contrast between norm salience and no norm salience in the MS condition, and *m* = 39 (36 studies) for the contrast between norm salience and no norm salience in the mortality-not-salient condition. For all comparisons, the polarity of the single effect size was chosen in accordance with the direction of compliance with the manipulated norm.

### Study-Level Moderators

The following potential moderator variables for the MS × Norm Salience interaction were examined for their between-study influence on effect sizes. The allocation to the categories can be found in Table 1. To additionally gain insights into the direct effect of norm salience, four of these moderators were further tested on their influence on the simple effect of norm salience without MS.

**Table 1.** Overview of All Included Studies, Their Characteristics, Moderator Codings, and Effect Sizes.

ID	Authors, year, study #	Total *N*	Manipulated norm	DV	Norm salience subtlety	Norm category	IV order	Data collection	MS control	Sample origin	Delay tasks	Research team	Norm salience control	Effect size *g*	*SE*
1	Abdollahi et al. (2010)	150	Consensus for vs. against martyrdom	Support for martyrdom	2	2	1	1	2	4	2	3	2	**0.64**	0.21
2	Arndt et al. (2009), Study 3^a^	100	Skin tone: pale vs. bronze	Tanning intentions	2	2	1	1	2	2	2	4	2	**1.28**	0.32
3	Chen et al. (2019), Study 1^b^	132	Karma vs. neutral	Excessive consumption	2	3	1	1	2	3	–	4	1	**0.53**	0.17
4	Chen et al. (2019), Study 3^a,b^	226	Karma vs. neutral	Excessive consumption	2	3	2	1	2	3	–	4	1	**0.57**	0.19
5	Courtney et al. (2021), Study 1	220	Individualism vs. collectivism	Health intentions	1	1	2	2	2	2	1	4	2	0.23	0.14
6	Courtney et al. (2021), Study 2	225	Individualism vs. collectivism	Vaccination intentions	1	1	2	2	2	2	1	4	2	**0.31**	0.13
7	Du & Jonas (2015), Study 1^c^	167	Modesty vs. neutral	Explicit self-rating	2	1	2	2	1	3	1	2	1	0.28	0.16
8	Du & Jonas (2015), study 2^c^	155	Modesty vs. competence vs. neutral	Explicit self-rating	2	2	2	1	2	3	1	2	1	0.26 –0.30	0.20 0.20
9	Fairlamb & Cinnirella (2021), Study 1^d^	149	Tolerance vs. neutral	Contact intention with Muslims	2	3	2	2	1	1	0	4	1	–0.07	0.20
10	Fairlamb & Cinnirella (2021), study 3^a^	243	Tolerance vs. neutral	Support of author rights	2	3	2	2	2	2	1	4	1	**0.45**	0.18
11	Fritsche et al. (2010), Study 1	83	Environmental norm: pro vs. anti	Liking of an eco-friendly car	1	1	2	1	2	2	2	2	2	0.41	0.22
12	Fritsche et al. (2010), Study 2^a^	101	Common interest vs. self-interest	Cut wood in a game	2	1	1	1	2	2	0	2	2	**0.46**	0.20
13	Fritsche et al. (2010), Study 3	107	Environmental norm: pro vs. neutral	Proportion of reusable cups	2	1	1	1	1	2	1	2	1	0.38	0.20
14	Gailliot et al. (2008), Study 1	45	Egalitarianism vs. neutral	Attitudes toward blacks	2	1	2	1	2	2	0	4	1	**0.61**	0.30
15	Gailliot et al. (2008), Study 2	57	Helping vs. neutral	Helping in scenarios	2	1	2	1	2	2	0	4	1	0.33	0.25
16	Gailliot et al. (2008), Study 3	108	Helping vs. neutral	Helping the confederate	2	1	–	3	1	2	0	4	1	**0.54**	0.20
17	Gailliot et al. (2008), Study 4	113	Social responsibility vs. neutral	Helping the confederate	1	1	–	3	1	2	0	4	1	0.37	0.19
18	Giannakakis and Fritsche (2011), Study 3	66	Individualism vs. collectivism	Allocation of resources	2	2	1	1	1	1	1	2	2	**0.64**	0.25
19	Greenberg et al. (1992), Study 2^e^	50	Tolerance vs. neutral	Evaluation of author	2	3	2	1	1	2	1	3	1	**0.55**	0.26
20	Harrison & Mallett (2013) ^a^	100	Environmental values vs. neutral	Collective eco guilt	2	3	2	1	2	2	0	4	1	**0.45**	0.20
21	Hirschberger et al. (2016), Study 1^a^	118	Justice vs. utility mindset	Support of violence	2	1	2	1	2	4	1	3	2	0.36	0.18
22	Hirschberger et al. (2016), Study 3^a^	339	Justice: high vs. low	Support of retribution	2	1	1	2	2	5	1	3	2	**0.25**	0.11
23	Hirschberger et al. (2016), Study 4^a^	90	Justice: high vs. low	Support of retribution	2	1	1	2	2	3	1	3	2	**0.49**	0.21
24	Jonas & Fritsche (2012)	72	Optimism vs. pessimism regarding winning	Estimated odds of winning	2	2	1	1	1	1	0	2	2	0.43	0.24
25	Jonas et al. (2008), Study 1^a^	77	Proself vs. prosocial norm	Attitude toward helping children	1	1	2	1	2	1	1	2	2	**0.71**	0.24
26	Jonas et al. (2008), Study 2	66	Pacifism vs. neutral	Pacifistic attitudes	1	1	2	1	2	1	1	2	1	**0.89**	0.26
27	Jonas et al. (2008), Study 3	76	Conservatism vs. benevolence	Bond for a prostitute	1	1	2	1	2	1	1	2	2	0.41	0.23
28	Jonas et al. (2008), Study 4	67	Helping vs. neutral	Helping (scenarios)	1	1	2	1	2	1	1	2	1	0.47	0.25
29	Jonas et al. (2013), Study 2	67	Generosity vs. neutral	Charity donation	1	1	2	1	2	2	2	2	1	0.48	0.25
30	Jonas et al. (2013), Study 3	74	Fairness vs. neutral	Keeping money	1	1	1	1	2	2	1	2	1	**0.51**	0.24
31	F. A. Martin (2020), dissertation^c^	384	Reciprocity in game: stingy vs. generous partner	Trust game	1	1	1	1	1	2	2	4	2	**–0.21**	0.10
32	McCabe et al. (2015)	114	Prototype: healthy eater vs. typical person	Healthy eating	2	2	1	3	2	2	1	4	1	**0.49**	0.19
33	Rothschild et al. (2009), Study 1^a^	138	Biblical compassionate values vs. neutral	Support for military force	2	3	1	1	2	2	1	3	2	0.32	0.16
34	Rothschild et al. (2009), Study 2^a^	90	Biblical compassionate values vs. neutral	Support for military force	2	3	1	1	2	2	1	3	2	**0.51**	0.18
35	Rothschild et al. (2009), Study 3^a,e^	120	Religiously labeled values vs. non-religiously labeled values	Anti-Western attitudes	2	3	1	1	2	4	1	3	2	**1.18**	0.20
36	Routledge et al. (2004), Study 2	75	Advertisement: tanning vs. neutral	Interest in tanning products	1	2	1	1	2	2	0	4	1	**0.50**	0.23
37	Schindler & Reinhard (2015a), Study 1	99	Honesty vs. neutral	How serious are “false alarms”	2	1	1	2	1	2	1	1	1	0.28	0.20
38	Schindler & Reinhard (2015a), Study 2	156	Honesty vs. neutral	Truth bias	2	1	1	2	1	1	1	1	1	**0.35**	0.16
39	Schindler & Reinhard (2015a), Study 3	81	Honesty vs. solidarity	Truth bias	2	1	1	1	2	1	1	1	2	**0.48**	0.23
40	Schindler & Reinhard (2015b), Study 1	75	Reciprocity (door-in-the-face technique) vs. neutral	Buying a newspaper	1	1	1	2	2	1	1	1	1	**0.48**	0.23
41	Schindler & Reinhard (2015b), Study 2	122	Reciprocity (door-in-the-face technique) vs. neutral	Amount of money	1	1	1	3	1	1	0	1	1	**0.38**	0.18
42	Schindler, Reinhard, et al. (2019), study 1^a^	157	Honesty vs. neutral	Dishonesty in a dice game	1	1	1	1	2	1	1	1	1	0.44	0.24
43	Schindler, Reinhard, et al. (2019), Study 2	313	Honesty vs. competition vs. neutral	Dishonesty in a dice game	2	1	1	1	2	1	2	1	1	**0.32** –0.08	0.14 0.14
44	Schindler et al. (2013), Study 1	69	Reciprocity vs. neutral	Amount of tip (scenario)	1	1	1	1	2	1	1	1	1	0.48	0.24
45	Schumann et al. (2014), Study 1	89	Religious prime (magnanimity) vs. neutral	Accessibility of revenge words	2	3	2	2	2	2	1	4	1	**0.46**	0.21
46	Schumann et al. (2014), study 2	113	Religious prime (magnanimity) vs. neutral	% of funds allocated to own group	2	3	2	2	2	2	1	4	1	**0.42**	0.19
47	Schumann et al. (2014), Study 3	103	Eye for an eye vs. turn other cheek vs. neutral	Endorsement of revenge	2	3	2	2	2	2	1	4	1	0.17 **0.97**	0.24 0.26
48	Vail et al. (2009)	91	Compassion vs. neutral	Preference Obama vs. McCain	2	3	1	1	2	2	2	3	1	**0.63**	0.21
49	Vail et al. (2019), Study 1^e^	79	Tolerance vs. neutral	Anti-Islamic attitudes	2	3	2	1	2	2	2	4	1	0.44	0.23
50	Vail et al. (2019), study 2^e^	396	Tolerance vs. neutral	Anti-Islamic attitudes	2	3	2	2	2	2	2	4	1	0.19	0.10
51	Fritsche et al. (2005), unpublished data^f^	75	Fair vs. unfair distribution	Ingroup bias	2	2	1	1	2	1	2	2	2	0.19	0.23
52	Schindler & Reinhard (2011a), Unpublished Data 1	115	Reciprocity (door-in-the-face technique) vs. neutral	Further study participation	1	1	1	1	2	1	1	1	1	0.02	0.19
53	Schindler & Reinhard (2011b), Unpublished Data 2	118	Reciprocity (door-in-the-face technique) vs. neutral	Buying newspaper (scenario)	1	1	1	1	1	1	1	1	1	0.05	0.18
54	Schindler & Reinhard (2011c), Unpublished Data 3	120	Reciprocity (door-in-the-face technique) vs. neutral	Donation probability	1	1	1	1	1	1	1	1	1	0.00	0.18
55	Schindler & Reinhard (2011d), Unpublished Data 4	68	Reciprocity (favor) vs. neutral	Given money in dictator game	1	1	1	1	1	1	1	1	2	−0.23	0.24
56	Schindler & Reinhard (2011e), Unpublished Data 5	116	Reciprocity (door-in-the-face technique) vs. neutral	Donation probability	1	1	1	2	1	2	1	1	1	0.22	0.19
57	Schindler & Reinhard (2011f), Unpublished Data 6	174	Reciprocity (door-in-the-face technique) vs. neutral	Donation probability	1	1	1	2	1	2	1	1	1	−0.11	0.15
58	Schindler & Reinhard (2011g), Unpublished Data 7	119	Reciprocity (door-in-the-face technique) vs. neutral	Donation probability	1	1	1	2	1	2	1	1	1	−0.24	0.19
59	Schindler & Reinhard (2015c), Unpublished Data 8^a^	323	Honesty vs. neutral	Dishonesty in a dice game	1	1	2	2	2	2	1	1	1	**−0.42**	0.16
60	Schindler & Reinhard (2015d), Unpublished Data 9^a^	318	Honesty vs. neutral	Dishonesty in a coin toss game	1	1	2	2	2	2	1	1	1	−0.05	0.16
61	Schindler & Reinhard (2015e), Unpublished Data 10	98	Honesty vs. neutral	Dishonesty in a coin toss game	2	1	1	1	2	1	1	1	1	0.10	0.20

*Note.* The effect size *g* refers to the interaction between MS and norm salience. Bold effects are significant with *p* < .05 according to our calculations. For three studies, it was possible to calculate two effect sizes for the two-way interaction. The polarity of effect sizes was chosen in accordance with the direction of the hypothesized pattern. *SE* = standard error. ID = identifier: Studies are labeled with consecutive numbers. If no study # is provided, the article contained only one single study. Norm salience: 1 = *subtle norm salience manipulation*, 2 = *explicit norm salience manipulation*. Norm category: 1 = *injunctive norm*, 2 = *descriptive norm*, 3 = *personal norm*. IV order: 1 = *MS manipulation first*, 2 = *norm salience manipulation first*. Data collection: 1 = *laboratory*, 2 = *internet*, 3 = *field*. MS control: 1 = *neutral topic in the MS control condition*, 2 = *aversive topic in the MS control condition*. Sample origin: 1 = *Europe*, 2 = *North America*, 3 = *Asian*, 4 = *Arab*, 5 = *Israel*. Delay tasks: number of delay tasks. Research team: 1 = *Schindler/Reinhard*, 2 = *Jonas/Fritsche*, 3 = *Pyszczynski*, 4 = *others*. Norm salience control: 1 = *two-way interaction includes a norm-not-salient condition*, 2 = *two-way interaction includes salience of two opposing norms*. Dashes in norm salience manipulation or manipulation sequence cells mean that the study could not be assigned to one of the categories.

a This study included an additional factor (within/between manipulation or measure of individual differences) to investigate a predicted moderation of the interaction between MS and norm salience interaction. Effect sizes for this study were calculated for the predicted condition/trait level. ^b^This study was only included for the interaction effect analyses because the interaction pattern was not theoretically specified. ^c^This study included an additional exploratory manipulation with no clear a priori prediction. Effect sizes for this study were therefore calculated across this factor. ^d^The threat manipulation in this study included terror salience as an additional threat condition. This condition was ignored because of the focus on generic MS manipulation. ^e^This study was only included for the interaction effect analyses because a buffering effect of MS was hypothesized in the norm-salient condition. ^f^The threat manipulation in this study included three additional conditions manipulating control threat. These conditions were ignored because of the focus on generic MS manipulation.

#### Subtlety of norm salience manipulation

Some researchers have proposed that norm salience may fail to influence outcomes when norm primes are exceedingly subtle. By contrast, they suggest that MS induction causes active search for cues of social and cultural norms, facilitating the detection of norm cues and explaining the effect of MS on norm compliance (Jonas et al., 2008, 2014). We thus wanted to examine the effect of how norm salience was manipulated (explicit vs. subtle). All norm salience manipulations that likely caused conscious thinking about the norm and/or stated that the norm is valued in a given group were coded as explicit manipulation. As a result, 25 effect sizes were coded as “subtle priming” and 39 as “explicit priming.” We also tested whether this factor moderated the simple effect of norm salience without MS.

#### Norm category

We relied on a broad conceptualization of norms, and this allowed us to include a larger number of studies. To investigate potential differences, we explored whether effect sizes depend on the norm category. We coded the respective norm-related concepts into three categories: injunctive norms (information about what should be done by a certain group) versus descriptive norms (information about what is commonly done by a certain group) versus personal norms (values that serve as guiding principles and self-expectations about for to behave). The coding criteria referred to how the concepts had been introduced and described in the articles. Independent of the assigned norm category, all included studies investigated the interaction hypothesis between MS and the salience of a norm-related concept. As a result, 42 effect sizes were coded as “injunctive norm,” seven as “descriptive norm,” and 15 as “personal norm.” We also tested whether this factor moderated the simple effect of norm salience without MS.

#### Order of manipulations

Another potential moderator is the sequence of MS and norm salience manipulations (MS first vs. norm salience first). If vigilance for relevant norms in a situation is triggered through MS (as proposed by Jonas et al., 2014), it is possible that the order of manipulations matters, such that larger effects occur when MS was manipulated first. As a result, 35 effect sizes were coded as “MS manipulation first” and 27 as “Norm salience manipulation first,” whereas two effect sizes could not be coded for any of the categories because a clear decision was impossible.

#### Data collection

Addressing the debate about data quality in online studies (especially via MTurk; Chmielewski & Kucker, 2019), we analyzed data collection (laboratory vs. internet vs. field) as a potential moderator. As a result, 40 effect sizes were coded as “laboratory” and 20 as “internet.” Four effect sizes were coded as “field studies.” We also tested whether this factor moderated the simple effect of norm salience without MS.

#### MS control topic

Burke et al. (2010) found the effect of MS to be independent of whether the control topic was aversive (e.g., uncertainty, dental pain) or neutral (e.g., watching TV). This suggested that the threat of death is qualitatively distinct and that TMT effects cannot be explained through mere aversion. To test whether this also holds for MS effects on defending salient norms, we analyzed the MS control topic (neutral vs. aversive) as a potential moderator. As a result, 18 effect sizes were coded as “neutral” and 46 as “aversive.”

#### Sample origin

Assuming that social norm compliance is more important in collective (e.g., Asian, Arabian) than in individualistic (e.g., European, North American) cultures, one could argue that collective cultures are more prone to norm salience effects. We therefore coded and analyzed sample origin (Europe vs. United States vs. Asian vs. Arabian vs. Israel) as a moderator. As a result, 21 effect sizes were coded as “Europe,” 33 as “North America,” six as “Asian,” three as “Arabian,” and one as “Israel.” We also tested whether this factor moderated the simple effect of norm salience without MS.

#### Delay

Burke et al. (2010) found the effect of MS to be stronger when there was a longer delay between the MS manipulation and the dependent measure. To test whether this also holds for the present data set, we analyzed the number of delay tasks (zero vs. one vs. two) as a potential moderator. As a result, 10 effect sizes were coded as “no delay task,” 41 as “one delay task,” and 11 as “two delay tasks,” whereas two effect sizes could not be coded for any of the categories because a clear decision was impossible.

#### Research team

Most included studies in the present research were published by three research teams (Schindler/Reinhard vs. Fritsche/Jonas vs. Pyszczynski vs. others). Given that researcher effects had been documented to show larger effects being observed by the “American team” (Yen & Cheng, 2013), we explored whether effect sizes vary across research teams. Coding referred to whether one of the “team members” was the author or co-author of the respective article. As a result, 19 effect sizes were coded as “Schindler/Reinhard,” 15 as “Fritsche/Jonas,” 9 as “Pyszczynski,” and 21 as “others.”

#### Salience of opposed norms

We included studies that manipulated norm salience. While some studies primed two opposed norms, other studies only primed one norm and used a norm-not-salient control condition. Given that the nature of the interaction for the first case suggests a larger effect size than the latter case, we examined whether effect sizes for the interaction effect included two opposed salient norms (vs. inclusion of a norm-not-salient control condition) as a moderator. As a result, 44 effect sizes were coded as “opposed norms salient: no” and 20 as “opposed norms salient: yes.”

### Statistical Analyses

Conventional meta-analytical techniques assume that effect sizes are statistically independent. Including multiple effect sizes stemming from multiple outcomes or comparisons per study violates this assumption (Lipsey & Wilson, 2001). In the present meta-analysis, three studies contained multiple treatment groups compared with a single control group. Here, two effect sizes of each study were included. To account for the dependency of the effect sizes, we used robust variance estimation (RVE; Hedges et al., 2010). Meta-analysis and meta-regression were conducted using the *robumeta* package for R (Fisher et al., 2017) with Wald tests provided by the *clubSandwich* package (Pustejovsky, 2020). The small-sample correction for degrees of freedom (Tipton, 2015) was applied. This approach fits a random-effects model, allowing the true effect size to vary from study to study, with weights adjusted for the dependency between effect sizes.

To estimate the variance of true effects, we computed τ to estimate the standard deviation of the true effect across studies. We also computed $I^{2}$ to describe the proportion of variance in the effect size across studies attributable to heterogeneity (J. P. T. Higgins et al., 2003).

#### Moderator analyses

To test for moderation of the effect size across studies, we employed mixed-effects RVE models. RVE allows testing of moderators while modeling dependence among predictors (moderators) and outcomes (effect sizes). Without clear theoretical predictions regarding the size and direction of moderator effects, our approach was necessarily exploratory.

#### Adjustments for publication bias

Unadjusted effect size estimates assume that hypothesis-supportive data are as likely to be recovered for meta-analysis as hypothesis-threatening data. Given that publication bias and researcher degrees of freedom are understood to be pervasive problems in psychological science (Bakker et al., 2012; Franco et al., 2014, 2016; Hesse, 2018; John et al., 2012), unadjusted effect sizes are likely to be overestimated (Kvarven et al., 2020).

Bias adjustments can improve performance relative to a meta-analysis that does not adjust for publication bias (Carter et al., 2019; McShane et al., 2016; Moreno et al., 2009; Simonsohn et al., 2014). In application, these methods successfully identified the overestimation of ego depletion effects, for example (Carter et al., 2015; Carter & McCullough, 2014). Although these adjustments tend to draw attention when the bias-adjusted effect is no longer significant, there is ample literature in which a significant effect remains after bias adjustment (e.g., Bücker et al., 2018; Hamilton et al., 2020; Ritchie & Tucker-Drob, 2018).

There are several tests and adjustments for publication bias. A summary of the bias adjustments used in this study can be found in the glossary in Table 2. Each adjustment technique relies on certain assumptions, and an understanding of these adjustments can aid in the interpretation of results, especially when results from different adjustments do not converge. We provide a detailed explanation of the adjustments below. We avoided some popular tools due to their poor performance: The trim-and-fill method (Duval & Tweedie, 2000) tends to adjust too little when bias is strong (Carter et al., 2019), and Fail-Safe *N* (Rosenthal, 1979) neither tests nor adjusts for the presence of bias.

**Table 2.** Glossary of Bias-Adjustment Techniques.

Adjustment technique	Summary	Reference
Egger’s test	Tests for small-study effects by regressing observed effect sizes against their standard errors. As standard error (sample size) does not cause effect size, no relationship is expected in the absence of publication bias. A negative slope indicates bigger effect sizes for smaller studies. This can be caused by publication bias: Small studies only reach statistical significance when they have overestimated the true effect size, whereas large studies can be published without such overestimation. In the absence of compelling reasons to expect bigger effects for smaller studies, a significant slope suggests evidence of publication bias.	Egger et al. (1997)
Sample-size Precision Effect Test (SPET)	Like Egger’s test, SPET relies on regression of effect size against a measure of study precision. It considers the intercept rather than the slope. This estimates the effect size that would be predicted from a linear extrapolation to a perfectly precise study (infinite sample size). This linear model assumes that all studies face equal publication bias regardless of their sample size. SPET tends to underestimate the size of non-null effects. A modified estimator is used for the standard errors to avoid downward bias.	Stanley & Doucouliagos (2013), Pustejovsky & Rodgers (2019)
Sample-size Precision Effect Estimate with Standard Errors (SPEESE)	SPEESE adopts the same approach as SPET, except that the extrapolation uses a quadratic, rather than a linear, relationship with study precision. This quadratic model assumes that publication bias is stronger among small studies, which must overestimate the effect to get published, and weaker among large studies, which are well-powered enough to avoid the file-drawer. SPEESE tends to overestimate the size of null effects. A modified estimator is used for the standard errors to avoid downward bias.	Stanley & Doucouliagos (2013), Pustejovsky & Rodgers (2019)
*p*-uniform	*p*-uniform estimates the true effect size using the distribution of *p* values for only those studies that produced a statistically significant result. When the null is true, the distribution of statistically significant *p value*s is expected to be uniform. When there is a true positive effect, the distribution of *p* values should be right-skewed, with more low *p* values than high *p* values. The extent of the right skew is proportional to the average statistical power of the studies, and the approach provides an estimate of the true effect that would yield that level of skew. It is fundamentally similar to *p*-curve.	van Assen et al. (2015)
Three-parameter selection modeling (3PSM) *p*-curve	This approach models publication bias with a parameter representing how much less likely a nonsignificant result is to be published than a significant result. The other two parameters represent the estimated bias-adjusted mean effect and the estimated heterogeneity of the effects. The *p*-curve is the plot of statistically significant *p* values. It uses the skewness of the distribution of significant *p* values to estimate the average study power. A right-skewed distribution suggests a true effect studied with some power. A flat distribution, by contrast, suggests that there is no evidential value. A left-skewed distribution suggests the exploitation of researcher degrees of freedom.	Hedges & Vevea (1996) Simonsohn et al. (2014)

##### Small-study effects

In the absence of publication bias and *p*-hacking, there is no relationship between the true effect size and the standard errors (i.e., direct function of sample size) of studies. Large-sample studies have small standard errors and cluster closely around the true effect size, and small-sample studies have large standard errors and are spread more diffusely around the true effect size. Because sampling error is symmetrical, studies of any size are as likely to *over*estimate the effect as to *under*estimate it. The relationship between effect size and the respective standard error is often displayed graphically as a *funnel plot* to show the spread of effect size estimates around the average effect size. Like sampling error, the funnel plot is expected to be symmetrical, with no correlation between sample size and effect size.

However, such a correlation can be observed if statistically significant studies are more likely to be published than nonsignificant studies (i.e., publication bias). Small-sample studies have large standard errors and only reach statistical significance when the observed effect size is large. Large-sample studies have small standard errors and can reach statistical significance when the observed effect size is small. Publication bias conceals studies from the lower-left portion of the funnel, creating *funnel plot asymmetry.* Significant asymmetry can be detected by testing the regression of effect size on standard error among the observed studies. *P*-hacking similarly creates small-study effects by nudging nonsignificant results toward the right side of the funnel where they become significant.

At the same time, there are benign causes that produce small-study effects other than publication bias (Page et al., 2021). When the true effect size varies across studies, it is possible that sample size and effect size are confounded with some third variable like methods, data quality, or population. For example, if some studies measure a large, obvious effect, and other studies measure a small, subtle effect, and both sets of studies are each appropriately powered, then a small-study effect will be observed. Misattributing this small-study effect to publication bias would result in overadjustment for publication bias and an underestimation of the true effect size. For this reason, it is important to explore possible confounds between sample size and effect size and to interpret small-study effects within the context of benign causes.

We present several tests and adjustments that consider these small-study effects. Egger’s test regresses effect sizes on the standard errors of the standardized effect sizes. A significant regression slope indicates a small-study effect. Sample-size Precision Effect Test (SPET) extrapolates from this regression slope to estimate the expected effect size of a hypothetical study with an infinite sample size. This performs well when the true effect size is approximately zero but can underestimate nonzero true effects. Sample-size Precision Effect Estimate with Standard Errors (SPEESE) fits a quadratic relationship between effect size and standard errors. This model assumes that publication bias is stronger among small-sample studies, but as sample size increases, studies reach a point that they are sufficiently powered and therefore experience little publication bias. This performs well when there is a nonzero true effect size but can overestimate null effects. Naturally, both regression models require substantial extrapolation, but they often provide better estimates than unadjusted meta-analysis or trim-and-fill (Carter & McCullough, 2014; Carter et al., 2019; Moreno et al., 2009).^3^

##### Selection modeling

An alternative approach to testing and adjusting for publication bias considers the *p* values rather than the effect sizes and sample sizes. When the null hypothesis is true, *p* values are uniformly distributed between 0 and 1. When the null hypothesis is false and studies have even a little power, *p* values have a right-skewed distribution: *p* values between 0 and .01 are more likely than *p* values between .04 and .05. As the average study power increases, this right skew becomes more pronounced. Selection models use the degree of skewness to estimate the power of studies and thus estimate the bias-adjusted true effect size.

In some cases, a *left* skew may be observed, such that *p* values between .04 and .05 are more common than *p* values less than .01. This phenomenon cannot be explained by the behavior of *p* values on their own; it is theorized that such a left skew is caused by the exploitation of researcher degrees of freedom (“*p*-hacking”) that move nonsignificant results until they are just-statistically significant (Simonsohn et al., 2014). Because this left skew can cancel out the right skew caused by study power, selection modeling methods can underestimate true effects in the presence of *p*-hacking.

Several methods exist for meta-analysis using the *p* values of results. We applied *p*-uniform (van Assen et al., 2015; as implemented in the *puniform* package, van Aert, 2019), the three-parameter selection model (3PSM; Hedges & Vevea, 1996; McShane et al., 2016; as implemented in the *weightr* package, Coburn & Vevea, 2019), and *p*-curve (Simonsohn et al., 2014; *p-curve app 4.06*). *p-*uniform uses the distribution of statistically significant *p* values to estimate the bias-adjusted effect. The 3PSM applies a similar method to all the *p* values, not just the statistically significant *p* values. This model attempts to estimate the average effect size, the degree of heterogeneity, and the degree to which nonsignificant results are less likely to be retrieved for meta-analysis. The *p*-curve uses the skewness of the distribution of statistically significant *p* values to estimate the average study power. The *p*-curve is the plot of statistically significant *p* values. A right-skewed distribution suggests a true effect studied with some power. A flat distribution, by contrast, suggests that there is no evidential value. A left-skewed distribution suggests the exploitation of researcher degrees of freedom.

Because these methods rely on the distribution of *p* values instead of small-study effects, they make a useful alternative model of publication bias. Importantly, they do not mistake benign small-study effects for publication bias. When there are large-sample small-effect studies and small-sample large-effect studies, these methods return the average power across both groups of studies. Thus, what can cause overadjustment in SPET and SPEESE will not cause overadjustment in *p-*uniform and 3PSM.

##### Applying adjustments for publication bias

The goal of bias adjustments is not necessarily to test for the presence of publication bias. Rather, the point is to try to estimate what the meta-analysis would report if one had all the data, published and unpublished. That said, it is best to give these adjustments all the data possible to improve the accuracy of this estimation (Carter et al., 2019). We therefore included the unpublished data. All analyses, it should be noted, include mostly published studies and are designed to work even if unpublished literature is unavailable. Nevertheless, we also report results for adjustments when excluding the unpublished data.

There is no single best bias-adjustment method. Furthermore, the results of different methods are not guaranteed to converge: Some adjustments perform well under certain conditions, and others perform well under different conditions. Thus, researchers should consider a variety of adjustments and interpret them according to their strengths, weaknesses, and model assumptions (Carter et al., 2019; Inzlicht et al., 2015; Kvarven et al., 2020; Sladekova et al., 2022; van Elk et al., 2015).

Among the methods we use here, SPET may be biased downward when the null hypothesis is false, and SPEESE may be biased upward when the null hypothesis is true. *p*-uniform does not model heterogeneity, and this can lead it to overestimate the average effect size of all studies (McShane et al., 2016). *p*-curve, *p*-uniform, and the three-parameter selection model (3PSM) assume that the decision to publish or not publish depends on the *p*-value of the meta-analyzed effect sizes; this can be misleading when one meta-analyzes simple effects, but the decision to publish hinges on a higher order interaction effect: If the interaction is required to be statistically significant (*p* < .05), at least one simple effect is likely to be highly significant (*p* < .01; Simonsohn et al., 2014). Applying selection modeling approaches like *p*-uniform or 3PSM to the simple effects may therefore substantially overestimate the true effect size. For that reason, we report bias-adjusted results only for the interaction effect. Funnel plots and bias adjustments for the simple effects can be found on the OSF.

Only some of these approaches are compatible with RVE. SPET and SPEESE can be applied alongside RVE as they are simple meta-regressions (Pustejovsky & Rodgers, 2019). However, *p*-uniform and selection modeling cannot be applied in the robust variance estimation framework because these methods assume one effect size per study. For these methods, it is not appropriate to average effect sizes together within studies because it is assumed that publication decisions are based on the *p* value of individual effects rather than the *p* value of the average of all effects. For these methods, we used bootstrapping. Where there are multiple dependent effect sizes within a study, we sampled one effect size at random from each study, then fit the model. This creates a set of independent effect sizes. The process was then repeated 500 times so that the results are representative of the broader set of possible random choices, rather than any one random selection of effects from within studies. For these methods, we reported the mean point estimate and mean confidence interval bounds from the 500 bootstraps.

## Results

### General Study Characteristics

The 61 included studies and the associated 64 effect sizes are presented in Table 1. Twelve of the 61 studies were not published (one was part of a doctoral thesis but not published in a peer-reviewed journal). In all, 44 studies included the typical MS manipulation (two open questions about death vs. a control topic), in seven studies participants were asked to write down the first sentence that comes to mind when thinking about one’s death, and one study used fliers with death-related content; nine studies applied other manipulations. Regarding the nature of the manipulated norms, 40 studies were coded as having included a salience manipulation of prosocial injunctive norms such as helping, charity, generosity, modesty, collectivism, egalitarianism, pacifism, benevolence, justice, fairness, reciprocity, and honesty. Further injunctive norms referred to pro-/anti-environmental norms, conservatism, and proself norms (individualism, competence, self-interest, or competition). Seven studies were coded as having included a salience manipulation of descriptive norms referring to skin tone, optimism/pessimism about winning, distribution of money, and support for martyrdom/violence; 14 studies were coded as having included personal norms referring to tolerance, karma, compassion, and magnanimity. Sample sizes of the 61 studies ranged from 45 to 396, resulting in an average sample size of about 134 participants per study (*SD* = 83.10; *Mdn* = 113).

### Main Analyses

**Figure 3.**
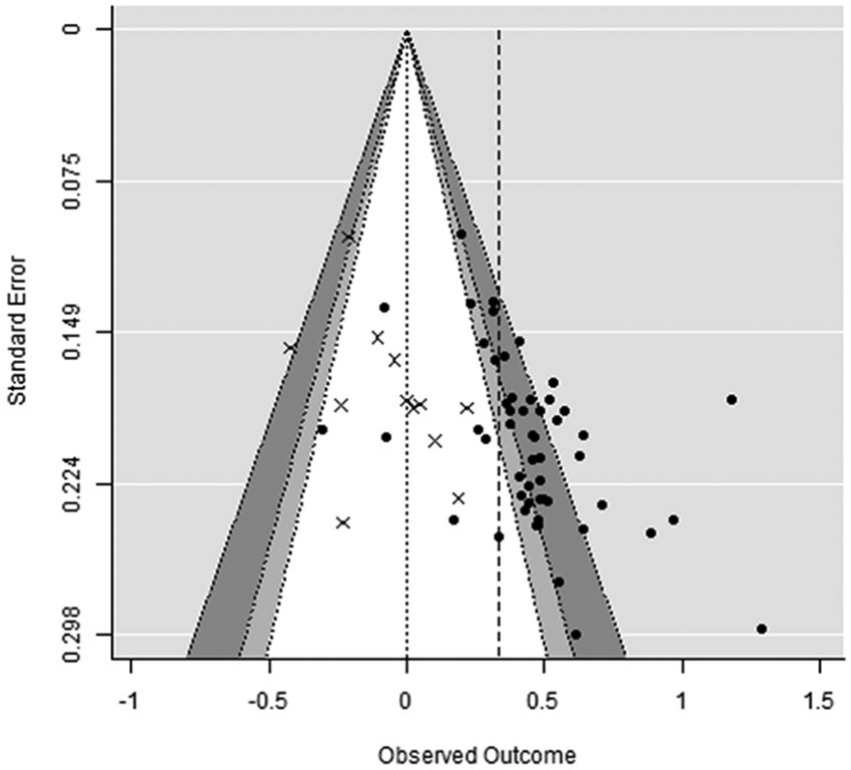
Funnel plot for the interaction effects between mortality salience and norm salience (m = 64). *Note.* Dependent effect sizes within three studies were averaged together for display purposes. Crosses represent unpublished data. The crude dashed line represents the average effect size. Shaded regions represent .10 > *p_two-tailed_* > .05 (light gray) and .05 > *p_two-tailed_* > .01 (dark gray).

Results of the interaction and the simple effects are presented in Table 3. A funnel plot for the interaction effects is presented in Figure 3. Funnel plots for the simple effects can be found on the OSF.

**Table 3.** Summary of Main Results and Adjustments for Publication Bias.

Effect	Test	*g*	*SE*	*t*	*df*	*p*	LL	UL
MS × Norm Salience interaction	RVE	0.34	0.04	8.68	58.38	<.001	0.26	0.41
Simple effects								
MS vs. no MS with norm salient	RVE	0.40	0.05	7.98	51.58	<.001	0.30	0.50
MS vs. no MS without norm salient	RVE	−0.19	0.06	−3.45	32.47	.002	−0.30	−0.08
Norm vs. no norm salient with MS	RVE	0.48	0.08	6.15	34.29	<.001	0.32	0.64
Norm vs. no norm salient without MS	RVE	−0.08	0.05	−1.62	31.79	.114	−0.18	0.02
Publication bias tests for the	SPET	−0.36	0.17	−2.14	13.71	.051	−0.71	0.00
MS × Norm Salience interaction	SPEESE	−0.03	0.10	−0.30	18.40	.771	−0.23	0.17
	*p*-uniform	0.15					0.01	0.27
	3PSM	0.05					−0.06	0.16

*Note.* RVE = Robust variance estimation; *g* = effect size; *SE* = standard error of *g; t* = *t* value associated with the *g* value in the same row; *df* = associated small-sample-corrected degrees of freedom; *p* = *p* value associated with the *t* value and *df* in the same row; CI = confidence interval; LL = lower limit of the 95% CI; UL = upper limit of the 95% CI.

#### The MS × Norm Salience interaction effect

On average, studies reported a significant MS × Norm Salience interaction, *g* = 0.34, 95% confidence interval [CI] [0.26, 0.41], *p* < .001. More than half of the variance in observed effect sizes was estimated to reflect true differences in effect sizes, τ=0.24 , *I*^2^ = 62.41%. According to common conventions, this amount of heterogeneity can be classified as moderate to substantial (J. P. T. Higgins et al., 2003). Meta-analyzing only the 49 published studies revealed a larger significant interaction effect, *g* = 0.43, 95% CI [0.37, 0.49], *p* < .001.

#### Simple effects

##### MS with and without a salient norm

On average, in conditions featuring a salient norm, MS increased norm-congruent outcomes, *g* = 0.40, *p* < .001, 95% CI [0.30, 0.50]. There was moderate heterogeneity, τ=0.27 , *I*^2^ = 53.62%.

On the contrary, in conditions not featuring a salient norm, MS did not increase but significantly decreased norm-congruent outcomes, *g* = −0.19, *p* = .002, 95% CI [–0.30, –0.08]. There was little heterogeneity, τ=0.12 , *I*^2^ = 25.06%.

##### Norm salience with and without MS

On average, in conditions featuring a MS manipulation, the presentation of a norm prime significantly increased norm-congruent behavior, *g* = 0.48, *p* < .001, 95% CI [0.32, 0.64]. There was moderate heterogeneity, τ=0.38 , *I*^2^ = 64.69%.

In the absence of a MS manipulation, however, the presentation of a norm prime did not significantly increase norm-congruent behavior, *g* = −0.08, *p* = .114, 95% CI [−0.18, 0.02]. There was little heterogeneity, τ=0.39 , *I*^2^ = 14.97%.

#### Moderation tests

We tested whether study-level features predicted the MS × Norm Salience interaction effect sizes observed in the studies. An overview of the results of the moderation analyses can be found in Table 4.^4^ As a quality check, we also report results when excluding the unpublished studies, given that most produced nonsignificant effects. Note that in these analyses power is reduced.

**Table 4.** Results of Study-Level Moderation Analyses.

Moderator	Summary effect and 95% CI							Test of moderation			Egger’s test
	*g*	LL	UL	*t*	*df*	*p*	*m*	*Statistic*	*p*	*I* ^2^	*p*
Subtlety of norm salience								*t*(50.0) = 2.87	.006	55.5%	
Explicit	0.42	0.34	0.51	10.54	33.8	<.001	39				.036
Subtle	0.20	0.07	0.34	3.09	23.0	.005	25				.015
Norm category								*F*(2, 14.8) = 5.41	.017	57.2%	
Injunctive norm	0.25	0.16	0.35	5.61	37.7	<.001	42				.002
Descriptive norm	0.56	0.31	0.80	5.63	5.8	.001	7				—
Personal norm	0.47	0.31	0.63	6.27	12.6	<.001	15				—
Order of manipulations								*t*(50.2) = 0.00	.997	63.8%	
MS first	0.33	0.22	0.45	5.96	32.3	<.001	35				.015
Norm salience first	0.33	0.22	0.45	5.89	23.2	<.001	27				.011
Data collection								*F*(2, 8.4) = 4.52	.047	61.1%	
Laboratory	0.40	0.29	0.50	7.60	36.0	<.001	40				.013
Internet	0.21	0.08	0.34	3.41	17.6	.003	20				.151
Field	0.45	0.32	0.58	10.92	3.0	.002	4				—
MS control topic								*t*(31.9) = 2.77	.009	57.7%	
Neutral	0.18	0.04	0.32	2.68	16.5	.016	18				—
Aversive	0.40	0.31	0.49	9.11	39.5	<.001	46				.002
Sample origin								*F*(4, 8.9) = 1.52	.276	60.5%	
Europe	0.30	0.17	0.42	4.90	18.6	<.001	21				.061
North America	0.32	0.20	0.43	5.77	29.8	<.001	33				.002
Asian	0.37	—	—	3.46	4.0	—	6				—
Arabian	0.72	—	—	2.97	2.0	—	3				—
Israel	0.41	—	—	—	—	—	1				—
Number of delay tasks								*F*(2, 17.3) = 0.53	.600	62.7%	
Zero	0.39	0.26	0.53	6.54	8.9	<.001	10				—
One	0.31	0.21	0.41	6.24	37.3	<.001	41				.012
Two	0.34	0.07	0.61	2.90	8.4	.019	11				—
Research team								*F*(3, 34.1) = 8.22	.007	52.4	
Schindler/Reinhard	0.11	−0.02	0.25	1.77	16.6	.095	19				—
Fritsche/Jonas	0.43	0.30	0.55	7.30	12.7	<.001	15				—
Pyszczynski	0.55	0.35	0.76	6.34	7.8	<.001	9				—
others	0.37	0.24	0.51	5.84	17.7	<.001	21				.021
Opposed norms salient								*t*(36.4) = 1.37	.180	62.5%	
No	0.30	0.21	0.39	6.88	39.1	<.001	44				.007
Yes	0.42	0.26	0.58	5.40	18.3	<.001	20				.104

*Note. g* = effect size; CI = confidence interval; LL = lower limit of the 95% CI; UL = upper limit of the 95% CI; *t* = *t* value associated with the *g* value in the same row testing statistical significance in the respective moderator subgroup; *df* = associated small-sample-corrected degrees of freedom; *p* = *p* value associated with the *t* value and *df* in the same row; *m* = number of effect sizes in the respective moderator subgroup. Statistic (test of moderation): *t* value for single parameter tests or *F* value for multiple parameter tests and according degrees of freedom. Significant test statistics indicate the significance of the overall model. *I*^2^ reflects the proportion of true variance in the total observed variance of effect sizes after accounting for the respective moderator. Note that for two subgroups in the *sample origin* analysis, degrees of freedom fell below 4. Significance tests for the summary effects should thus not be interpreted. Accordingly, we did not report LL, UL, and *p* values for the respective subgroups. For one subgroup in the *sample origin* analysis, there was only one study; therefore, significance tests for the summary effects were not conducted. Egger’s test refers to the positive relationship between the effect size and the standard error. A significant *p* value indicates a significant relationship suggesting small-study effects and an overestimation of the unadjusted effect size. Egger’s test was only conducted for moderator subgroups including 20 studies or more. All tested relationships were positive in direction; nonsignificant effects should be interpreted with caution due to low test power.

##### Subtlety of norm salience manipulation

On average, effect sizes were significantly larger when explicit norm manipulations were used (*g* = 0.42) than when norm manipulations were subtle (*g* = 0.20), *t*(50.0) = 2.87, *p* = .006. The moderation was no longer significant when excluding the unpublished data, *t*(22.9) = −0.19, *p* = .851.

##### Norm category

The difference among outcomes of the three norm categories was significant, *F*(2, 14.8) = 5.41, *p* = .017, with descriptive norms producing the strongest effect (*g* = .56) compared with personal norms (*g* = 0.47) and injunctive norms (*g* = 0.25). The moderation was no longer significant when excluding the unpublished data, *F*(2, 12.3) = 3.01, *p* = .086.

##### Order of manipulations

When norm salience was manipulated prior to MS (*g* = 0.33), effects were practically equal compared with when MS was manipulated first (*g* = 0.33), *t*(50.2) = 0.00, *p* = .997. This moderation approached significance when excluding the unpublished data, *t*(39.9) = −1.73, *p* = .091, indicating that larger effects occurred when MS was manipulated first (*g* = 0.48 vs. 0.37).

##### Data collection

The differences among outcomes of the three data collection formats was significant, *F*(2, 8.4) = 4.52, *p* = .046, with data collection in the lab (*g* = .40) or in the field (*g* = 0.45) producing a stronger effect compared with internet studies (*g* = 0.21). The moderation was still significant when excluding the unpublished data, *F*(2, 6.1) = 83.1, *p* < .001, with data collection in the lab (*g* = 0.49) or in the field (*g* = 0.44) producing a stronger effect compared with internet studies (*g* = 0.32).

##### MS control topic

Significantly larger effects occurred when the MS control condition referred to an aversive topic (*g* = 0.40) compared with a neutral topic (*g* = 0.18), *t*(31.9) = 2.77, *p* = .009. The moderation was no longer significant when excluding the unpublished data, *t*(15.4) = 1.42, *p* = .177.

##### Sample origin

Five types of sample origin were coded. Over half of the effect sizes came from studies using participants from North America (*g* = 0.32), followed by studies using participants from Europe (*g* = 0.30). A lower number of studies included Asian participants (*g* = 0.37) and Arabian participants (*g* = 0.72). One study included participants from Israel (*g* = 0.41). Despite the substantially larger effect size found in Arabian samples, the overall analysis between the subgroups was not significant, *F*(4, 8.9) = 0.52, *p* = .276. Excluding the unpublished data also revealed no significant moderation effect, *F*(4, 8.2) = 0.34, *p* = .842.

##### Delay

Descriptively larger effects occurred when no delay task was applied after the MS manipulation (*g* = 0.39) compared with one task (*g* = 0.31) or two tasks (*g* = 0.34). However, the overall analysis between the subgroups was not significant, *F*(2, 17.3) = 0.53, *p* = .600. Excluding the unpublished data also revealed no significant moderation effect, *F*(2, 14.0) = 0.25, *p* = .783.

##### Research team

The overall difference between the outcomes of the four research team categories was significant, *F*(3, 28.6) = 6.58, *p* = .002, with “Schindler/Reinhard” being the only team where no significant effect occurred (*g* = 0.11), *p* = .096. Effect sizes of “Fritsche/Jonas” (*g* = 0.43), “Pyszczynski” (*g* = 0.55), and “others” (*g* = .37) were all significant, all three *p*s < .001, and significantly larger than the effect sizes of “Schindler/Reinhard,” all three *p*s < .007. The moderation was no longer significant when excluding the unpublished data, *F*(3, 20.2) = 1.17, *p* = .348.

##### Opposed norms salient

On average, effect sizes were descriptively larger when the norm salience manipulation applied two opposed norms (*g* = 0.42) compared with when a norm-not-salient control condition was employed (*g* = 0.30). This difference was, however, not significant, *t*(36.4) = 1.37, *p* = .180. Excluding the unpublished data also revealed no significant moderation effect, *t*(29.7) = 1.43, *p* = .164.

##### Moderators of norm salience effect without MS

To gain insights into the direct effect of norm salience, four of the above-analyzed moderators were further tested on their influence on the simple effect of norm salience without MS. The four moderators referred to the subtlety of the norm salience manipulation, norm category, data collection, and sample origin. None of the factors yielded a significant moderation effect, all four *p*s > .095. Excluding the unpublished data also revealed no significant moderation effects, all four *p*s > .336.

#### Adjustments for publication bias

Results of the bias adjustments for the interaction effect across the whole data set are presented in Table 3.

##### Egger’s regression test

Egger’s regression test is a meta-regression of effect sizes on standard errors. This regression showed a significant relationship between standard errors and effect sizes, *b* = 3.56, *t*(16.1) = 4.41, *p* < .001, 95% CI [1.85, 5.28], such that smaller studies found larger effects.

##### SPET/SPEESE

SPET similarly regresses the effect sizes on their standard errors. By following this regression line to its intercept, one receives an estimate of what the effect sizes might be at a study with zero standard error, removing the publication bias indicated by the small-study effect. The intercept was negative and almost significant, *g* = –0.36, 95% CI [–0.71, 0.002], *p* = .051, suggesting the true effect is small and overestimated due to publication bias.

SPEESE regresses the effect sizes on the square of their standard errors. The resulting quadratic curve allows bias to be strong among small studies and weaker among large studies. The slope of this curve was statistically significant, again indicating small-study effects, *t*(19.2) = 4.41, *p* < .001. Like SPET, the intercept of this model estimates the true effect. This estimate was not significant, *g* = –0.03, 95% CI [–0.23, 0.17], *p* = .771, again suggesting that the true effect is small and overestimated due to publication bias.

Applying SPET only to published studies again showed a significant relationship between standard errors and effect sizes, *b* = 2.58, *t*(8.7) = 6.14, *p* < .001, 95% CI [1.63, 3.54], and reduced the average interaction effect to nonsignificance, *g* = –0.07, 95% CI [–0.26, 0.12], *p* = .407. SPEESE also suggested small-study effects, *t*(12.9) = 5.3, *p* < .001, but revealed a small, significant effect, *g* = 0.16, 95% CI [0.04, 0.28], *p* = .015.

##### p-uniform/3PSM

The *p*-uniform adjustment yielded a smaller but still statistically significant effect, *g* = 0.15, 95% CI [0.01, 0.27]. 3PSM adjusted the effect size estimate down, arriving at a small nonsignificant effect *g* = 0.05, 95% CI [–0.06, 0.16]. The adjusted model fit significantly better than the unadjusted model, χ^2^(1) = 36.55, *p* < .001, indicating significant selection bias for significant results over nonsignificant results. The adjusted model estimated that *p* values greater than .05 were 5%, 95% CI [0%, 11%], as likely to be published as *p* values less than .05.^5^

Applying 3PSM only to published studies again revealed a small, significant effect, *g* = 0.18, 95% CI [0.06, 0.30]. Again, the adjusted model fit better than the unadjusted model, χ^2^(1) = 36.78, *p* < .001. The adjusted model estimated that *p* values greater than .05 were 5% [0%, 10%] as likely to be published as *p* values less than .05. Note that excluding unpublished studies did not change *p*-uniform because all the unpublished studies have *p* > .05, and *p*-uniform only considers studies with *p* < .05.

##### *p*-curve

Finally, we conducted a *p*-curve analysis for the MS × Norm Salience interaction. Where possible, we took the relevant test statistic directly from the article text. Where that was impossible (7 cases), we transformed our scraped effect sizes into a *t*-test by dividing the effect size by its standard error. As recommended, we included only one test statistic per study. For the three studies providing two effect sizes, we took the test reported first in the manuscript. We further excluded two effect sizes that were significant in the opposite direction. As a result, we included 59 test statistics, of which *p*-curve uses only the 41 statistically significant statistics.

**Figure 4.**
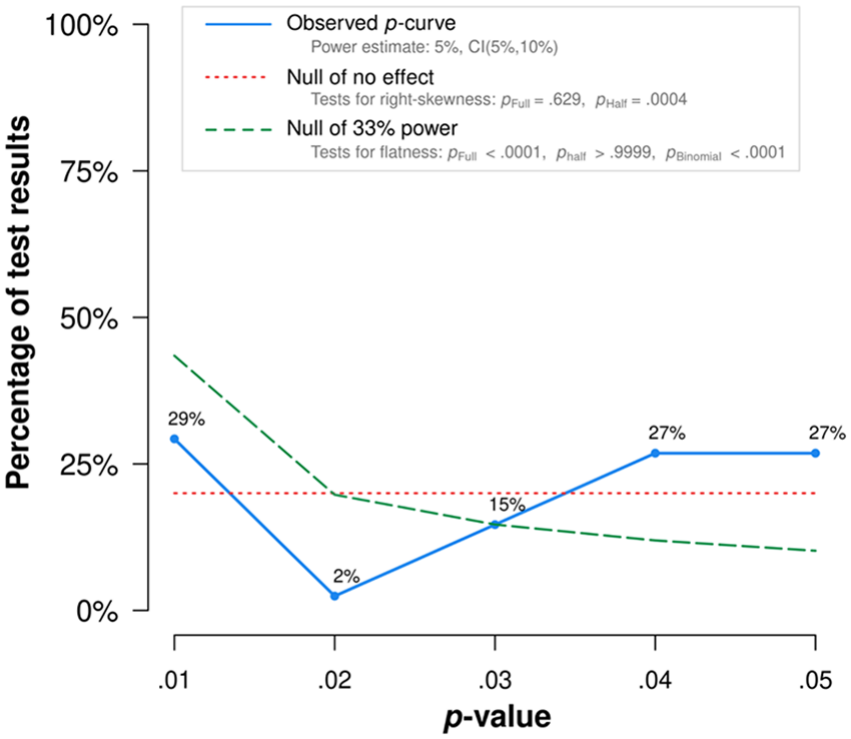
Results of a *p*-curve analysis on published test statistics of interaction effects between mortality salience and norm salience. *Note.* The observed *p*-curve includes 41 statistically significant (*p* < .05) results, of which 16 are *p* < .025. There were 18 additional results entered but excluded from *p*-curve because they were *p* > .05.

In the full *p*-curve, there was no significant right skew, *p* = .629, indicating no evidential value of the submitted effects. Instead, the full *p*-curve had a distinct left skew, with 54% of the reported *p* values between .03 and .05 (Figure 4). Estimated power for the submitted effects was 5%, 95% CI [5%, 10%], significantly less than 33% power, *p* < .001. To address the possibility of “ambitious *p*-hacking,” that is, exploitation of researcher degrees of freedom to reach *p* values substantially below .05, Simonsohn et al. (2015) proposed a *p*-curve of only *p* values less than .025 (i.e., in our case 16 *p* values). This “half *p*-curve” was proposed as a more conservative test for evidential value, as it includes fewer studies and is less likely to mistake ambitious *p*-hacking for evidential value. In the half *p*-curve, the test for right-skewness was significant, *p* < .001, indicating evidential value; in this test, power was not significantly less than 33%, *p* > .999.

##### Small-study effects in subgroups of moderators

Small-study effects are expected to reflect publication bias. However, it is possible that small-study effects may instead represent a moderator variable that is correlated with both sample size and effect size, creating an artifactual small-study effect. To examine this possibility, we tested for small-study effects within moderator subgroups. To ensure ample power, we only applied the test in subgroups with at least 20 studies. Results of the significance test for the positive relationship between the effect size and standard error can be found in Table 4. All 14 tested relationships were positive in direction and 11 were significant. In three subgroups, the tests were not significant with *p*s < .151; here the subgroups only included 20 or 21 studies, respectively. These results indicate that small-study effects can be detected in most moderator subgroups, suggesting that effect sizes are likely overestimated within each moderator subgroup and that the small-study effect is not likely to be explained by any of the current moderators.

The subgroup of lab studies deserves a closer look. The moderator analyses above revealed that the average effect size was twice as large when data were collected in the lab compared with internet studies. One possibility is that this indicates reduced treatment and measurement quality in internet studies. As the average online sample size (*n* = 185) was substantially larger than that of the average lab sample size (*n* = 110), this suggests an alternative explanation for the small-study effects we observed: Could it be that large-sample online studies have smaller true effects than small-sample lab studies, potentially due to decreased manipulation strength or measurement quality online? The tests for small-study effects in the subgroup of lab studies suggest otherwise: In this subgroup of 40 effect sizes, Egger’s test detected a small-study effect such that smaller studies found larger effects, *b* = 3.80, *t*(6.9) = 3.30, *p* = .013, 95% CI [1.07, 6.53]. In addition, SPET and SPEESE both reduced the average effect size of the interaction to nonsignificance, although the confidence intervals did not exclude small-to-medium effects (SPET: *g* = –0.39, *p* = .168, 95% CI [–1.01, 0.22]; SPEESE: *g* = –0.01, *p* = .947, 95% CI [–0.37, 0.35]).

## Discussion

Considering the number of differing and opposing reactions to MS (e.g., helping vs. aggression), TMT has been criticized for being unfalsifiable because any finding could be interpreted post hoc as evidence in favor of the theory. One answer to this challenge is that reactions to MS depend on the situational salience of social norms. The present work aimed to assess the empirical evidence for this idea by focusing on studies that manipulated both MS and norm salience. Given that social norms define the *cultural* part of one’s worldview, our analysis refers to the original cultural worldview defense hypothesis of TMT.

### Basic Findings

We meta-analyzed data from 61 studies. Regarding the interaction effect between MS and norm salience, across 64 effect sizes, our meta-analysis revealed a significant medium effect size (*g* = 0.34). To further examine the nature of the interaction effect, we analyzed four simple effects. Most importantly from a TMT perspective, the results revealed a significant medium effects size for MS versus no MS, given a salient norm (*g* = 0.40). The contrast for MS versus no MS without a salient norm was also statistically significant but in the reversed direction (*g* = –0.19). The results further revealed no significant effect for norm-salient versus norm-not-salient without MS (*g* = –0.08). The contrast between norm-salient and norm-not-salient yielded a significant medium effect under MS (*g* = 0.48). Overall, this pattern is in line with the proposed MS × Norm Salience interaction hypothesis.

### Moderation Analyses of the Interaction Effect

Investigating moderators is a potential jumping-off point for future theoretical and empirical work. In the following, we summarize the main results of the moderation analyses and then discuss the implications and limitations.

Some researchers have proposed that norm salience may fail to influence outcomes when norm primes are exceedingly subtle (e.g., Jonas et al., 2008). In line with this notion, interaction effects were larger for explicit compared with subtle norm salience manipulations. As a possible explanation, subtle norm cues might be more often overlooked by participants. Alternatively, simply reminding people of a social virtue, such as benevolence, might not be a sufficiently specific manipulation of a social norm. This is because virtues are not always relevant to social norms. Instead, it needs the perception of social consensus within a personally and situationally relevant social in-group about holding, and acting in line with, a specific value or rationale. Explicit norm manipulations might provide such consensus information.

The interaction effect was further significantly moderated by norm category: Effect sizes were larger when norm salience referred to descriptive or personal norms compared with injunctive norms. This again supports the previous reasoning that effective norms must be *relevant*. They become relevant when people either personally internalize them or consider their group to act upon them, not just giving lip service.

Effects were significantly larger in lab studies than in internet studies. One interpretation is that this indicates lower data quality in internet studies (Chmielewski & Kucker, 2019). Additional bias analyses suggested that the larger effect sizes in lab studies were likely due to small-study effects (i.e., sample size) as a confound, in the sense that the smaller studies (which were more likely conducted in the lab) resulted in larger effects. This might be due to more extensive *p*-hacking and publication bias in the lab compared with the internet studies. At the same time, it could indicate higher quality (e.g., strength of the specific manipulations) of the lab studies.

Significantly larger effects occurred when the MS control condition referred to an aversive topic compared with a neutral topic. This may point to the value of holding affect constant when investigating the effects of existential threats. As previous research by Lambert et al. (2014) shows, conscious affect following MS manipulations may have independent (and sometimes contrary) effects on anxiety buffer measures that may conceal specific existential threat effects. For instance, negative (vs. neutral) affect may elicit more elaborate processing, and this may counteract MS effects.

Researcher teams produced different effect sizes with “Schindler/Reinhard” being the only team with no significant effect. It could be argued that the team “Schindler/Reinhard” shows a lack of researchers’ “intuitive flair for how to set up the most conducive situation and produce a highly impactful procedure” (Baumeister, 2016, p. 157). However, given that almost all unpublished studies came from “Schindler/Reinhard,” the researcher team was no longer a significant moderator when excluding the unpublished data. In addition, recent investigations failed to show evidence for the claim that researchers’ expertise and diligence generally produce larger or more precise effect sizes and higher replication success (Ebersole et al., 2020; Protzko & Schooler, 2020). Thus, the reasons why these unpublished studies did not produce significant effects remain speculative.

No significant moderation effects occurred for order of the MS and norm salience manipulations, sample origin, number of delay tasks, and whether there was an opposed norm salience condition. However, applying two opposed norms resulted in noticeably larger effects, as expected.

Taken together, the moderator analyses might be interpreted as a prompt to use explicit norm salience manipulation, to manipulate MS prior to norm salience, to collect data in the lab, and to use a norm salience manipulation with two opposed norms (see recommendations for future research below). However, the significance or nonsignificance of any of these moderators should be considered with caution, as we have conducted a plethora of significance tests, and so the moderator analyses should be viewed as exploratory. In addition, we applied Egger’s test for each moderator subgroup to check for small-study effects and possible overestimation of the unadjusted effects. All tested relationships between effect sizes and sample sizes were positive, and nearly all tests were significant. These findings qualify clear recommendations of any particular study features for inferring the specific boundary conditions of the interaction effect or making the interaction effect easier to replicate in future studies.

### Adjustments for Publication Bias

Publication bias is a pervasive problem in psychological science (Bakker et al., 2012; Franco et al.,2014, 2016; John et al., 2012; Rosenthal, 1979), and only recently have scientific reforms attempted to reduce it (Hesse, 2018). Thus, the question is not whether publication bias exists in a certain body of literature, but to what degree it affects the published evidence and what effect sizes might be expected after adjustment for that bias. Unfortunately, despite our efforts to search for unpublished data, we were unable to include any unpublished work, except for 11 studies we conducted and one external doctoral dissertation. Given that unadjusted effect size estimates are based on the assumptions that there is zero publication bias and zero use of researcher degrees of freedom, looking at the bias adjustments seems mandatory. We applied several tests to detect and adjust for potential publication bias in the gathered literature. Results consistently indicated overestimation of the “true” interaction effect, although they differed in their estimates of the effect size.

#### Small-study effects

Egger’s regression test indicated that smaller studies found larger interaction effects; this was also the case for nearly all subgroups of the investigated moderators. SPET and SPEESE also rely on the association between effect sizes and study precision and reduced the average interaction effect to nonsignificance, with 95% CIs excluding effects of *g* = 0.17 and larger.

Readers may wonder about the SPET estimate being negative and nearly significant. Does this method mean to suggest that there is a true effect in the opposite direction? If so, can anything be learned from the SPET estimate when it is so likely to be wrong? SPET and SPEESE have a downward bias in the presence of *p*-hacking (Carter et al., 2019), and negative and statistically significant SPET estimates have been observed in both simulation studies (Carter et al., 2019) and meta-analysis (Hilgard et al., 2019). Inspection of the funnel plot suggests that the regression slope is likely to be pulled by the small studies with very large effect sizes (e.g., Arndt et al., 2009, *g* = 1.28) and a large study with a significant negative effect (Schindler & Reinhard, 2015c, unpublished study 8, *g* = –0.42). There may also be downward bias inflicted by *p*-hacking (Carter et al., 2019). Rather than interpreting the SPET estimate as indicating a negative true effect, it would be more appropriate to infer that there is a small or null true effect and that SPET is yielding an underestimate, whether due to sampling error, influential small-sample and large-sample studies, or *p*-hacking.

SPET and SPEESE assume there is no correlation between true effect size and sample size. Importantly, this assumption may be challenged in the case that large studies have smaller true effect sizes than small studies. In this case, SPET and SPEESE may underestimate effects if there is a benign cause for the small-study effect (e.g., Page et al., 2021). Some scholars have expressed concerns about the quality of measurement or efficacy of manipulation in large-sample research, expecting small-sample research to yield larger effects due to more intense and effortful manipulations and a more impactful procedure (Baumeister, 2016). In the existing literature, lab studies have smaller sample sizes compared with internet studies, so this confounding between data collection and sample size may be a benign explanation for the observed small-study effects. We tested this alternative explanation and still found small-study effects within lab studies alone. Finally, *p*-uniform and 3PSM rely on the distribution of *p* values, not small-study effects. Therefore, they do not experience downward bias in the presence of benign small-study effects. Nevertheless, both of those adjustments still estimated a smaller effect (see below).

Although small sample sizes do not guarantee greater quality of measures and manipulations, small studies might sometimes indeed be the more effortful and thus effective ones (e.g., when they involve personal interactions between participants and the experimenter). We therefore caution against attributing these small-study effects solely to publication bias as there may be other unknown benign causes for the negative relationship between sample size and effect size (Page et al., 2021).

#### Selection models

*p*-uniform indicated a statistically significant, but small, interaction effect with 95% CIs excluding effects of *g* = 0.27 and larger, whereas 3PSM reduced the interaction to nonsignificance. In addition, 3PSM achieved a significantly better model fit when adjusting for bias, indicating that studies with nonsignificant effects are less likely to be included in this meta-analysis, even after the inclusion of our own unpublished work.

#### *p*-curve

The half *p*-curve of originally reported *p* values associated with the MS × Norm salience interaction indicated significant right skew and substantial statistical power, meaning that for studies reporting *p*s < .025 evidential value is present and that the null hypothesis of no effect can be rejected. However, the full *p*-curve was markedly left-skewed, indicating no evidential value. At the same time, the average study power for the interaction effect was estimated at between 5% and 10%, significantly <33%. That is, the evidence is not absent but inadequate, meaning that the existence of small effects cannot be ruled out.

On the one hand, this may be an underestimate of average study power—in simulation studies, *p*-hacking adds large *p* values to the *p*-curve, canceling out the right skew and biasing the *p*-curve estimate downward (Carter et al., 2019). On the other hand, this could also be an overestimate of average study power, as heterogeneity in effect sizes can cause the *p*-curve to overestimate mean power (Brunner & Schimmack, 2020; McShane et al., 2016). We observed moderate heterogeneity among effect sizes, and we cannot know to what degree, if any, the collected test statistics are affected by *p*-hacking. Therefore, it is difficult to know whether the *p*-curve estimates are accurate, biased upward, or biased downward. The findings of the *p*-curve should therefore be interpreted with caution.

#### Summary of adjustments

Results of bias adjustments consistently reduced the effect and suggest low statistical power, but there is still a lack of convergence as some techniques show a significant effect, but others do not. This demonstrates that such techniques on their own may not be sufficiently robust to firmly establish the presence or absence of an effect (Inzlicht et al., 2015; Kvarven et al., 2020). What is needed is a good faith attempt to show the basic effect in high-quality studies (see recommendations for future research below; van Elk et al., 2015).

### Conceptualization of Social Norms

#### Inclusion of studies

By referring to injunctive, descriptive, and personal norms, we relied on a broad conceptualization of social norms. This decision implied a challenge when assessing the eligibility of relevant articles because several judgment calls had to be made regarding whether a study included a social norm manipulation. In an earlier version of this manuscript, we used a narrower scope and limited inclusion to articles explicitly using the term “social norms.” In this version, we meta-analyzed data from 30 studies and 32 effect sizes (most of the articles on personal norms were not included). The interaction effect between MS and norm salience revealed a significant small-to-medium effect *g* = 0.34, 95% CI [0.24, 0.45]. All bias adjustments estimated the effect size as nonsignificant (range *g*_corrected_ = –0.38 to 0.04). The full *p*-curve of the interaction effects was noticeably left-skewed, suggesting possible exploitation of researcher degrees of freedom.

In contrast to this narrow approach, we additionally applied a more liberal perspective: We meta-analyzed a broad set of studies by also including studies using priming manipulations that have only a tenuous link to social norms (i.e., presence of others, autonomy, heroes, global climate change, shared human experience, humanizing violence, popular celebrities, stereotypes, ingroup responsibility for the victim, and adversary intent). Across these 89 studies and 92 effect sizes, the interaction effects between MS and norm salience revealed a significant small-to-medium effect *g* = 0.38, 95% CI [0.32, 0.45]. Bias-adjustment techniques arrived at smaller effect size estimates for the hypothesized interaction, sometimes reducing the effect to nonsignificance (range *g*_corrected_ = –0.10 to 0.24). The *p*-curve indicated that the evidential value of the submitted effects is inadequate due to the low statistical power of the studies. More detailed results can be found on the OSF. In sum, these findings basically mirror the picture of the findings presented in the main result section above: There is a significant small-to-medium effect, while bias adjustments suggest a smaller or sometimes even no effect. We are therefore confident that our conclusions are not altered by whether a study aimed to explicitly address the social norm construct. Nevertheless, even the explicit social norm studies may have fallen short in properly manipulating social norms, given that many just made general social virtues salient instead of manipulating perceived consensus regarding beliefs and behavior in self-relevant groups. Future work needs clear assumptions and conceptualization about what constitutes a social norm (see recommendations for future research below).

#### Heterogeneity

Although most included studies featured similar manipulations of MS, manipulations of social norms varied widely, activating different norms through different methods. Moderator analyses of norm category suggested larger effects for descriptive and personal norms (vs. injunctive norms). It is possible that some of the between-study heterogeneity we observed is caused by differences in the personal or collective relevance of different norms; future research may investigate this as a potential moderator.

### Lack of Evidence for Social Norm Priming

In the absence of MS, there was no effect of norm primes. This result is not in line with predictions of the focus theory of normative conduct (Cialdini et al., 1991; Reno et al., 1993) but does correspond to several studies showing no norm prime effects on norm conformity (e.g., Bergquist & Nilsson, 2016, study 1; Hamann et al., 2015; Kallgren et al., 2000, study 1; Lawrence, 2015), providing a further data point that decreases confidence in the validity of social norm priming effects—at least without taking into account boundary conditions. These results, however, may be influenced by publication bias (the same holds for the reversed significant simple effect of MS when no norm was salient). As mentioned above, if study results are selected for a significant MS × Norm Salience interaction, that will select for the simple effect of norm salience to be large in the MS condition and the simple effect of norm salience to be small (or even reversed) in the mortality-not-salient condition. Therefore, publication bias may be actually *underestimating* the effects of norm salience given no MS.

Some authors argued that missing effects of norm salience on norm compliance might be due to the use of subtle primes (e.g., Fritsche et al., 2010; Jonas et al., 2008), but our moderation analysis of the subtlety of the norm salience manipulation does not support this notion. It should be said that previous research often used primes in the form of direct appeals, such as “please do not litter” (Cialdini et al., 1990) or “please don’t remove the petrified wood from the park” (Cialdini et al., 2006). None of the included studies in our meta-analyses used such explicit manipulations. Beyond subtlety of the norm salience manipulation, we also tested norm category, data collection, and sample origin as moderators. None was significant.

In addition, people should not always be motivated to follow the salient norm. Instead, people may contradict norms when they feel pressured to conform (see research on reactance; Miron & Brehm, 2006), do not identify—or even disidentify—with the reference group or conventional society (e.g., Giannakakis & Fritsche, 2011), want to stress individuality after experiences of depersonalization (Brewer, 1991), or perceive injunctive and descriptive norms to contradict each other (Smith et al., 2012). On the contrary, people may conform to social norms when they are highly identified with the reference group (Masson & Fritsche, 2014; Reinhard et al., 2014; Schindler et al., 2016; Terry et al., 1999), are personally uncertain (Smith et al., 2007), experience a threat to self-esteem (vanDellen et al., 2011), or to control (Stollberg et al., 2017). As a first indication that norm priming effects might be contingent on motivational state, Kallgren et al. (2000) found these effects only when physiological arousal or self-focus were increased. Thus, simple norm activation might often be insufficient, and additional motivational factors may be important to increase norm conformity. The proposed MS × Norm Salience hypothesis suggests that one such factor could be confrontation with one’s own mortality (Jonas et al., 2008). In line with this reasoning, our meta-analysis revealed significant moderate norm salience effects when mortality was salient, but not when mortality was not salient.

### Recommendations for Future Research

Unadjusted results of our meta-analyses suggest that MS and norm salience interact to influence behavior. At the same time, the results of bias adjustments reveal uncertainty about the size and the robustness of the effect. What is the way forward? Considering the present findings and the issues already mentioned, we briefly discuss recommendations for future work—concerning both theoretical and methodological aspects—to establish the necessary conditions to reliably show the effect and to advance the research (Fiedler, 2017; Muthukrishna & Henrich, 2019; Oberauer & Lewandowsky, 2019).

#### Boosting theorizing

Falsifiability needs theory specification (Glöckner & Betsch, 2011). TMT has been criticized for being unfalsifiable because any finding could be interpreted as evidence in favor of the theory (L. L. Martin & van den Bos, 2014). Pyszczynski et al. (2015) admit that it is often difficult to a priori predict exactly how people will respond to MS and that more precise elucidation is needed. Assuming social norms as a crucial aspect of the cultural worldview, the present idea that MS guides people’s reactions according to the social norm that is momentarily salient specifies boundary conditions for the MS effect and addresses the criticism of non-falsifiability because reversed findings are evidence against TMT (i.e., if MS would increase reactions opposed to the salient norm). To further increase theory specification, we discuss the underlying motives behind norm conformity under MS.

##### Relevance of social norms for the worldview and self-esteem

According to TMT, the MS × Norm Salience hypothesis is built on the assumption that MS increases people’s need for the protection furnished by a valid cultural worldview and by the perception of living up to this worldview (self-esteem). Thus, for MS to guide normative behavior, it is necessary for the salient social norm to be perceived as an important aspect of people’s cultural worldview and to provide a source of self-esteem. Accordingly, researchers primed concepts that they thought would represent a perceived important norm for participants (e.g., honesty or generosity). However, none of the included studies empirically tested this assumption, so it is possible that some of the studies did not manipulate norms that have clear relevance for participants’ cultural worldview and their self-esteem.

For some of these norms, whether they are actually endorsed by majorities or by the salient ingroup (e.g., anti-environmental norms or competition) might prove controversial. Thus, simple reminders of what researchers suppose people to perceive to be an important norm in their cultural group might not be sufficient, or may even prove counterproductive, in eliciting norm salience effects. Instead, manipulating participants’ perception of what the ingroup norm actually is (e.g., “82% of students share a norm of generosity”) might be more effective in eliciting strong norm conformity effects. Only a small minority of studies has addressed these issues and provided a direct manipulation of the social norm. For instance, Jonas and Fritsche (2012) manipulated the descriptive norm of optimism about the performance of people’s national soccer teams by directly providing alleged information about betting odds at betting agencies.

The existing varying personal relevance of the primed norm further highlights the role of individual differences measures. In one study, Harrison and Mallett (2013), for example, found that the MS × Norm salience interaction effect on experiencing environmental guilt only occurred for people who strongly endorsed pro-environmental values. Whereas our meta-analysis is limited to situational (i.e., manipulated) norm salience, such assessments may reflect self-esteem relevance and chronic accessibility of cultural worldviews.

##### Anxiety-buffering function of groups

We addressed the role of social norms as a fundamental part of cultural worldviews, meaning that these norms are shared by most of a certain group. Instead of concentrating on the worldview concept as the heart of the anxiety buffer, some terror management researchers have focused on the role of group membership and affiliation as possible anxiety buffers. Castano and colleagues (Castano & Dechesne, 2005; Castano et al., 2002) have proposed group membership, or social identity, as an independent anxiety buffer because it provides a way for individuals to distance themselves from the parts of their personal identity that are going to die and to define the self in terms of an entity that continues to exist even in case of personal death (i.e., the social group). Supporting this idea, MS was shown to increase identification with salient ingroups (Castano et al., 2002; Fritsche et al., 2008; Giannakakis & Fritsche, 2011; Jonas & Fritsche, 2012; Sani et al., 2009). Similarly, Wisman and Koole (2003) found that participants under MS sat closer to other group members even if those group members held worldviews that were different from their own. The authors concluded that, at times, affiliation defenses and the need for social acceptance seem powerful enough to override worldview validation defenses. On the grounds of a social identity version of terror management, MS should increase people’s self-stereotyping in terms of those attributes and behaviors that they consider prototypical (i.e., normative) for their ingroup (Giannakakis & Fritsche, 2011; Halloran & Kashima, 2004). As an approximation, those MS studies that manipulated descriptive ingroup norms (e.g., Jonas & Fritsche, 2012) should be more suitable for testing this hypothesis than those that primed general norms or universal values of human conduct, such as benevolence (Jonas et al., 2008) or honesty (Schindler et al., 2019). However, attributes appear normative not only when ingroup members share them with each other but also when they do *not* share them with members of other groups (Turner et al., 1987). Thus, properly manipulating ingroup norms does not only require inducing consensus but also distinctiveness from outgroup norms. Finally, understanding cultural worldview defense as an expression of people’s social self (i.e., social identity; Castano et al., 2002) implies that as a response to MS, people will only defend the perceived norms of a group with whom they identify. That is, future studies should manipulate in-group norms and should ensure that all participants are chronically identified with that group to a sufficient degree (e.g., English students) and that the group is highly salient in the study situation (e.g., by announcing a study comparing English with Scottish students; e.g., Giannakakis & Fritsche, 2011).

As an alternative social identity–inspired approach, group-based control theory (Fritsche, 2022) predicts that people increasingly act on the collective level of their self (i.e., act in terms of ingroup norms; Stollberg et al., 2017) to restore a sense of control after their personal-level control has been threatened (e.g., due to MS; Fritsche et al., 2008). Given alternative models of terror management processes, future research should use more specific conceptualizations to address the underlying motives of MS-induced norm conformity (worldview bolstering, self-esteem striving, social self-stereotyping, or group-based action).

Further elaborating the dynamics of ingroup norm conformity may help clarify why MS × Norm salience effects might sometimes be fragile. As mentioned earlier, some norms primed in the analyzed studies might have contradicted people’s representation of the dominant norm in their group or culture. Research on “loyal deviance” (Packer, 2008; Packer et al., 2013) has found that people who are highly identified with their group deviate from norms contradicting ideal or injunctive in-group norms (Masson & Fritsche, 2018). If part of terror management motivation centers around strengthening social identity, for instance, then such loyal deviance processes may explain why in-group norms are at times not more strongly adhered to, or even violated, following MS. This should be particularly true when studies did not induce “pro-social” norms but norms of selfishness (Jonas et al., 2008, Study 1; Fritsche et al., 2010, Study 2), individualism (Giannakakis & Fritsche, Study 3), competition (Schindler et al., 2019, Study 2), or littering (Fritsche et al., 2010, Study 1), as these might have contradicted people’s idea about how their cultural ingroup should act (i.e., ideal or injunctive norm). In a similar vein, salient norms that obstruct the positive image of the in-group, such as pessimism about future ingroup performance (Jonas & Fritsche, 2012), might not be followed under conditions of MS, because people may want to protect and validate their cultural ingroup.

##### Summary of the necessary theoretical conditions

For MS to guide people’s reactions according to the social norm that is momentarily salient, it is necessary for the social norm to be perceived as an important aspect of people’s cultural worldview and to provide a source of self-esteem. That is, the social norm must be prevalent in a (salient) ingroup or culture with whom people identify. Given that almost all the pertinent studies have used non-validated norm concept primings and did not directly manipulate norm perception, the present body of studies may underestimate the size of the true interaction effect of MS and norm salience (this also applies to the found null effect of social norm priming without MS). In addition, as mentioned above, there is a broad variance in how social norms have been conceptualized. To increase falsifiability, future research needs to engage in theory specification by providing a finite set of construct definitions and propositions that together constitute their theory. Formal modeling can contribute to this aim (Glöckner & Betsch, 2011; Smaldino, 2017).

#### Boosting methodology

Given that most MS studies in TMT research did not manipulate norm salience, one could ask why many studies still found worldview defense of norms and values after MS, whereas our meta-analysis revealed even a significant negative effect of MS when no norm was salient. As Pyszczynski et al. (2015) mention in their review of TMT research, considering the complexity of people’s cultural worldviews and the interplay with *always present* situational factors, MS effects are never pure main effects. Thus, a main effect of MS on norm adherence does not necessarily contradict the MS × Norm Salience interaction hypothesis. It may be, for example, that in certain studies that did not explicitly manipulate norm salience, there was a main effect of MS on worldview defense, because of the chronic salience of certain worldviews and collective affiliations in the sample (such as patriotism of U.S. Americans). At the same time, group-based changes in what is considered the appropriate in-group norm may explain nonreplications of MS effects (e.g., on derogating critics of the United States; Chatard et al., 2020).

In other cases, the assessment of the dependent measure itself may unintentionally have functioned as a norm priming. The present reversed effect of MS when no norm was salient may be due to a variety of salient (and potentially opposing) worldview aspects in this condition—canceling each other out or even leading to a reversed effect when averaged. Thus, it becomes evident that more specific theorizing enables more precise operationalizations and more adequate empirical testing (Scheel et al., 2021). In the following, we discuss methodological aspects that need to be addressed in future research to gain scientific progress.

##### Validation of material

The present body of studies mostly lacks validation of the used material. For an adequate and informative test of the MS × Norm salience interaction hypothesis, a *valid* norm salience manipulation is needed (see Fiedler et al., 2021). This first requires knowledge about participants’ worldviews (Schindler et al., 2021) and whether the investigated social norms are perceived as relevant and in line with the majority of a salient ingroup. According to previous research, one such social norm could be honesty, given that the rule of not lying can be seen as universal (Schindler et al., 2021; Schindler, Reinhard, et al., 2019). At the same time, if taking a social identity perspective on TMT, those norms should be most identifiable as ingroup norms on which the ingroup markedly differs from a salient outgroup (e.g., when people of a collective culture compare with people from a group known to be individualistic; Halloran & Kashima, 2004). Thus, depending on which “version” of TMT a researcher accepts, priming honesty as a universal norm might be a good idea (people defend deeply ingrained cultural worldviews) or less so (people defend their collectives). That is, in addition to just relying on perceived consensus, perceived intergroup distinctiveness of a norm might be taken into account and pretested (e.g., making an outgroup salient that is stereotyped as being markedly less honest compared with the ingroup, for instance, scientists vs. marketing people).

In the next step, it should be ensured that the priming procedure increases the accessibility of the social norm compared with a control group, for example, by using word-fragment completion tasks or implicit association tests to measure accessibility. According to the results of our moderator analyses, using explicit primes (e.g., “Be honest!”) may be more effective than subtle ones (such as word-search puzzles containing words associated with the norm). The same applies to additionally priming an opposed norm (e.g., “Be rich!” or “Be the best!”) compared with using a neutral control condition.

Another issue concerns the experimental manipulation of MS. In our meta-analyses, 72% of the included studies used the classic two open-ended, short-answer questions that ask participants to write about the emotions that the thought of their own death arouses in them and to jot down what will happen to them as they physically die. We recommend planning the study design according to the classic worldview defense paradigm (i.e., classic MS manipulation, delay, dependent measure; see Schindler et al., 2021). To our knowledge, TMT remains silent about whether participants should deal with the MS manipulation in a certain way to trigger worldview defense reactions. For example, analyses of the time participants spent in the experimental conditions and of the quality of their answers did not moderate the null finding of the MS manipulation (Schindler et al., 2021, preregistered Study 3). Such analyses are typically not done in MS research. In fact, to our knowledge, participants’ answers to the MS questions have never been systematically analyzed. This is surprising because analyzing and categorizing these answers might lead to theoretical development and potentially to crucial moderators (Kastenbaum & Heflick, 2011). It seems plausible, for example, that participants who write about being sad leaving their family behind might react differently compared with participants writing that they do not care about their own death because different concepts are activated. Thus, investigating the classic MS manipulation itself and being specific about the related auxiliary assumptions to make the manipulation work may be a fruitful endeavor (see also Trafimow, 2022).

##### Powering the interaction

In the present work, we investigated the interaction between two factors. The *p*-curve analysis suggests that with an estimated power of 5%, the present body of studies was clearly underpowered to find the hypothesized interaction effect. In the following, we show how a priori power analyses for the investigated interaction may proceed. It is important, however, to emphasize that higher power in terms of a larger sample size alone is not enough to gain research progress—power and sample size always need to be interpreted within the theoretical and methodological context (Sassenberg & Ditrich, 2019).

Given the lack of convergence of the bias adjustments, for the following power analyses, we use the unadjusted estimated effect size of our meta-analysis for the MS × Norm Salience interaction, which is *g* = 0.34. Accordingly, an a priori power analysis in G*power (Faul et al., 2009) reveals a total sample size of *N* = 366 needed to detect effect sizes larger than *g* = 0.34 (analysis of variance [ANOVA]: main effects and interactions; *f* = 0.17 [equals *g* = 0.34]; α = .05; Power = .90; numerator *df* = 1; number of groups = 4). The *median* sample size among the present body of included studies was *N* = 113. Using this sample size for a post hoc power analysis revealed a power of about 43% to detect effect sizes larger than *g* = 0.34. However, it is important to recognize that we not only want to test for a significant interaction but that we expect a certain pattern of means and therefore want to test one or more simple effects as well. Therefore, we should consider the pattern of the expected interaction, as this has major implications for a priori power analyses (Blake & Gangestad, 2020; Giner-Sorolla, 2018; Lakens & Caldwell, 2021).

To provide a guideline for future research, we refer to the reasoning of Giner-Sorolla (2018) and briefly discuss the case of reversed interaction effects. In a perfectly reversed interaction (also called “disordinal interaction” or “cross-over interaction”), two equally large simple effects occur, and they are in opposite directions. In this case, the effect size of each of the simple effects equals the effect size of the interaction (Giner-Sorolla, 2018). For the MS × Norm Salience interaction, such a pattern can be theoretically assumed when applying priming of two opposed norms. For example, MS (vs. no MS) increases pro-environmental behavior when a pro-environmental norm is primed and increases anti-environmental behavior when an anti-environmental norm is primed. Most of the included studies in our meta-analysis applied priming of two opposed norms. As outlined above, whereas with *N* = 366 we can detect an interaction of *g* = 0.34 with 90% power, we have only about 63% power for the simple MS effect in the norm salience condition (calculated with R package *Superpower*; Lakens & Caldwell, 2021). To detect the simple effects with 90% power, we therefore must double the sample size that we calculated for the interaction, resulting in *N* = 732 (Giner-Sorolla, 2018). With the *median* sample size of the included studies of *N* = 113, we have only 24% power for each of the simple effects.

A reversed interaction including two equally large simple effects is not an unrealistic, but clearly an optimistic, scenario in the present context. Studies that implemented priming of two opposing norms rarely led to equally large simple effects of MS; more frequently, only one significant simple effect occurred with the other one being close to zero (i.e., attenuated interaction). Such changes in the interaction pattern may erratically increase the needed sample size (we refer to such attenuated interactions and lower effect sizes on the OSF).

##### Preregistration

Bias-adjustment results suggest the existence of publication bias, small effects, and/or exploitation of research degrees of freedom. We therefore caution researchers that MS × Norm Salience interactions may be smaller and more difficult to attain than a naïve reading of the literature would suggest. On the contrary, bias corrections may also underestimate the true effect due to questionable research practices. To counteract such practices, preregistration of studies is a powerful tool, but only two studies in our data set did so (Courtney et al., 2021, Study 2; Vail et al., 2019, Study 2). We therefore emphasize the need for detailed preregistrations of future studies in this area.

### Practical Implications

Considering that the news and social media often present themes of death, war, and terror attacks, the MS × Norm Salience hypothesis highlights the impact of such reporting on the behavior and the attitudes of recipients: When people are confronted with mortality, their subsequent reactions might depend on the prevalent norms. This also points to the influence that political or spiritual leaders could have on the opinion of their populations in presenting what is a valued reaction. Social norms can thus have an impact on the escalation or de-escalation of violent intergroup conflicts (Jonas & Fritsche, 2013): If norms of intragroup cooperation are established within a society, this could prevent these conflicts instead of catalyzing them as a consequence of MS. However, given the uncertainty about the size and robustness of the demonstrated effects, more research is needed to evaluate the validity of these competing ideas and to give policy recommendations.

## Conclusion

The present work aimed to assess the empirical evidence for the interaction effect between MS and social norm salience. Unadjusted results of the meta-analysis yielded a significant small-to-medium effect size. Subsequent simple effect analyses confirmed the hypothesized pattern of the interaction. Results of bias adjustments suggest that there is publication bias and/or the exploitation of researcher degrees of freedom, and these adjustments suggest smaller effect size estimates for the hypothesized interaction, sometimes reducing the effect to nonsignificance. It is possible, though, that use of researcher degrees of freedom has caused the adjustments for publication bias to underestimate the true effect size. Thus, the degree to which the bias-corrected estimates are biased themselves is unknown, and results should be interpreted with caution. The lack of convergence when using multiple bias-correction techniques is further problematic and demonstrates that such techniques alone may not be sufficiently robust to firmly establish the presence or absence of an effect.

The suggested existence of publication bias and potential use of researcher degrees of freedom call for improved methods such as using preregistration, collecting larger samples, using validated norm salience manipulations and standardized measures as outcomes, and committing to publishing both significant and nonsignificant results. At the same time, uncertainty about the size and robustness of the demonstrated effects should inspire more specific theorizing about the nature of cultural worldview defense and the motivational function of norm conformity under conditions of existential threat. We hope that this meta-analysis will motivate researchers to address these challenges.

## Notes

1 We decided to exclude two eligible studies published by Renkema et al. (2008). Although this 2008 article has not been officially retracted, one other paper with Renkema as first author and Stapel as co-author had been retracted due to fraudulent data supplied by Stapel. In addition, more than 50 articles in which Stapel was involved have been retracted (according to retractionwatch.com).

2 Standardized measures of effect size, like the Cohen’s *d* or Hedges’ *g* used in this manuscript and commonly throughout meta-analysis, standardize for population variance. The effect size is the difference between the means of each group divided by the pooled standard deviations of each group: *d* = (mean1—mean2)/(pooled *SD*). When the variance of scores is high, the denominator increases, and Cohen’s *d* shrinks (i.e., the difference between groups is small relative to the differences within groups). When the variance of scores is small, the denominator decreases, and Cohen’s *d* grows (i.e., the difference between groups is large relative to the differences within groups). Furthermore, the standard error of the standardized effect size is a direct function of the sample size.

3 To remove an artifactual correlation between effect sizes and their standard errors, we replace the original Precision Effect Test (PET) and Precision Effect Test with Standard Errors (PEESE) estimators (Stanley & Doucouliagos, 2013) with the modified SPET and SPEESE estimators (Pustejovsky & Rodgers, 2019).

4 We also conducted a meta-regression that analyzed the relation between publication year and effect size. Results yielded a significant negative association, *b* = −0.07, *t*(10.8) = −2.26, *p* = .046, indicating that effect sizes decreased with time. Hypothesizing that this effect may be attributable to larger sample sizes, we included the standard error of effect sizes as predictor. As a result, the association between publication year and effect size was reduced to nonsignificance, *b* = 0.01, *t*(12.9) = 0.14, *p* = .889, although there was a significant negative relation between sample size and effect size, *b* = 3.60, *t*(12.1) = 3.69, *p* = .003. This suggests that the decrease in effect size across years is confounded by an increase in sample size, making it difficult to determine whether the true effect size is shrinking or the degree of publication bias is shrinking.

5 An initial attempt at *p*-uniform and 3PSM analyses used the extracted effect sizes and standard errors. In this analysis, *p*-uniform adjustment reduced the effect to *g* = 0.28, 95% CI [0.14, 0.41]. The effect was also reduced by 3PSM, *g* = 0.23, 95% CI [0.11, 0.35], again finding better fit with the adjusted than the unadjusted model, χ^2^(1) = 4.50, *p* = .034, and estimating that *p* values greater than .05 were 39% [5%, 73%] as likely to be published as *p* values less than .05. Closer inspection of these analyses revealed that nine effect sizes were reported as having just-statistically significant *p* values in the original articles but were not significant by our calculations using reported means and *SD*s (these *p* values range between .051 and .092). These differences may be caused by errors introduced through rounding, transformation among effect sizes, or adjustments for effects and covariates, for which our scrape did not adjust. Because *p*-uniform and 3PSM rely on studies with large *p* values for their downward adjustment, the omission of these large-*p* studies may underestimate the degree of publication bias and overestimate the effect size. To fix this, we used the reported test statistic, effect size, and sample size to solve for the standard error that would yield the reported *p* value.
